# Antibiotic-altered gut microbiota explain host memory plasticity and disrupt pace-of-life covariation for an aquatic snail

**DOI:** 10.1093/ismejo/wrae078

**Published:** 2024-05-30

**Authors:** Gabrielle L Davidson, Ignacio A Cienfuegos, Sarah Dalesman

**Affiliations:** School of Biological Sciences, University of East Anglia, Norwich, NR4 7TJ, United Kingdom; Department of Psychology, University of Cambridge, Cambridge, CB2 3EB, United Kingdom; School of Biological, Earth and Environmental Sciences, University College Cork, Cork, Ireland; Department of Life Sciences, Aberystwyth University, Aberystwyth, SY23 3DA, United Kingdom; Department of Life Sciences, Aberystwyth University, Aberystwyth, SY23 3DA, United Kingdom

**Keywords:** gut microbiota, Lymnaea stagnalis, cognition, memory, behavioural plasticity, pace-of-life, syndromes, personality, antibiotic pollution, microbiome–gut–brain axis

## Abstract

There is mounting evidence that intestinal microbiota communities and their genes (the gut microbiome) influence how animals behave and interact with their environment, driving individual variation. Individual covariation in behavioural, physiological, and cognitive traits among individuals along a fast–slow continuum is thought to arise because these traits are linked as part of an adaptive pace-of-life strategy. Yet paradoxically, trait intercorrelation is absent or disrupted in some populations but not others. Here, we provide experimental evidence from aquatic pond snails *(Lymnaea stagnalis*) that environmental stressors and the gut microbiota explain host phenotypic plasticity and disrupted covariation among traits. Antibiotic exposure at varying levels of ecologically relevant concentrations had multiple effects starting with gut microbiota diversity, differential abundance, and inferred function. Memory declined in line with antibiotic concentrations that caused the most profound gut microbiota disruption, and although pace-of-life traits remained rigid, their covariation did not. Moreover, inferred microbial metabolic pathways with biologically relevant host functions explained individual and treatment variation in phenotypes. Together, our results point to the gut microbiome as a proximate mechanism influencing the emergence and maintenance of phenotypic variation within populations and highlights the need to decipher whether the gut microbiome’s sensitivity to environmental pollution facilitates adaptive or maladaptive phenotypic plasticity.

## Introduction

It is clear from experiments with lab rodents that alterations to their gut microbiota generate changes in behaviour and cognition [1]—the mental processes that allow animals to store and act on information from their environment. Although the gut microbiome has the potential to transform our understanding of behavioural variation and plasticity in natural populations [2], progress in this field is lacking [3]. Behaviour facilitates rapid flexible responses to changing environments. Yet even under shared, fluctuating environmental conditions, populations are polymorphic where individuals vary consistently in behaviours such as boldness, with risk-taking individuals on one extreme of a continuous behavioural axis and risk-averse individuals on the other (personality, [4]). Different behavioural traits, including boldness and exploration, commonly correlate positively among individuals (behavioural syndromes, [5]) and, in some cases, are positively correlated with metabolic rate [6], and linked either positively or negatively to individual cognition, such as learning speed and memory accuracy [7, 8].

A leading hypothesis states that behavioural, physiological, and life history traits may covary because of pleiotropic genetic effects or common physiological pathways sensitive to current environmental states [5, 9–11]. Therefore, consistent, covarying individual differences in behaviours and cognition arise because they represent different pace-of-life strategies (pace-of-life syndromes, [5, 7, 11]). However, phenotypic covariation is not ubiquitous across organisms, and why covariation is detected in some populations or species but not others remains an unresolved question in animal ecology and evolution [8, 12]. Disparities in the patterns of covariation across systems may reflect differential trait sensitivities to environmental stressors [13], requiring phenotypic plasticity. The gut microbiome is theorized to be the missing link, driving host cognition, behaviour [2], metabolic rate [14], and pace-of-life syndromes [15]. The consequences of gut microbiome perturbation in natural populations are necessary to test these hypotheses, but are rare [16–18], in part due to limitations in microbiome interventions that are in keeping with an animal’s natural ecology [3, 19]. Pond snails (*Lymnaea stagnalis*) are an established model system for population and individual variation in cognition, and pace-of-life traits [20–22] where covariation and plasticity are habitat- and context-specific, and likely associated with environmental stressors [22, 23]. We predicted that individual variation and plasticity could be mediated by the enteric microbial community and their collective genes, given the compelling evidence from clinical rodent research demonstrating causal and mechanistic evidence that the gut microbiome regulates host biological processes and phenotypes [1, 24–26].

Environmental contamination from widespread antibiotic use can alter the gut microbiome of aquatic and soil organisms [27], which we predicted could have additional downstream effects to host phenotypes and their covariation. We exposed wild-sourced, F2 generation aquatic pond snails (*L. stagnalis*) to a mixture of two broad-spectrum antibiotics—sulfamethoxazole (SMX) and oxytetracycline (OTC)—dissolved in their aquarium at representative levels reported in freshwater ecosystems across the globe [28–30]. We evaluated the impact of increasing concentrations of antibiotics on the microbial community by exposing snails to four different treatments (low, medium, high, and a sham control) in a between-subject design. We reported whether these different treatments altered the relative abundance, diversity, and inferred function of the gut microbiota [31]. The antibiotic exposures additionally served as a tool to manipulate the gut microbiota at differing intensities to test the gut microbiota’s role in shaping cognition and pace-of-life traits including exploration behaviours, metabolic rate, and their covariation.

## Materials and methods

### Antibiotic exposure

Snails were placed in groups of three individuals into closed 1.8-L-capacity aquaria to remove effects of social isolation [23] for 72 h prior to phenotypic assays (see below). A 72-h incubation time was selected following previous pharmaceutical exposures that were sufficient to cause nonlethal physiological effects in *L. stagnalis* [32, 33]. Aquaria were sterilized and filled with 0.4L of UV-sterilized oxygenated artificial pond water per snail (1.2 L in total). SMX and OTC were solubilized in dimethylsufoxide (DMSO) at a final maximum solvent concentration of 0.0033% (concentration in the 4 μg/L antibiotic exposure group and DMSO control group). SMX was chosen because it is of particular concern in the UK and elsewhere due to its high consumption and discharge rate, and OTC is highly persistent and nonbiodegradable [34, 35]. We did not sample the Sowy river where the lab stock of snails was sourced and therefore did not test for SMX or OTC contamination, although these antibiotics have been reported in UK rivers generally [35–38]. Final sample sizes phenotyped across treatment groups included: a control group (standard pond water, *n =* 22, in 13 aquaria), and three antibiotic exposures where SMX and OTC were each at the following concentrations and in the same solution: low: 1 μg/L (26 individuals, in 12 aquaria), medium: 2 μg/L (27 individuals, in 12 aquaria), and high: 4 μg/L (32 individuals, in 15 aquaria). To ensure DMSO had no effect on phenotypes, we tested 29 individuals exposed to DMSO at 0.0033% only representing the highest DMSO exposure in the 4-μg/L antibiotic exposure group and compared phenotypes to the pond water control group using linear mixed models (LMMs). As described below, there was no significant difference in: memory: *t* = 0.51, *P =* .61, thigmotaxis: *t* = −0.98, *P =* .33, speed: *t* = −1.26, *P =* .22, metabolic rate: *t* = 1.68, *P =* .11.

### Phenotypic assays

To test the effects of antibiotics and the gut microbiome on snails, we measured a suite of traits that are commonly considered part of pace-of-life phenotypes [5, 11]. We performed well-established assays described in this system for each snail in the following order: memory [22], thigmotaxis [23], speed [23], and metabolic rate [39].

### Long-term memory

We tested long-term memory formation in snails following a classical conditioning assay. The expectation is that snails should form a negative association between food (the unconditioned stimulus) and an aversive stimulus (potassium chloride, KCl), and therefore when presented with carrot juice after a delay (memory retention interval), they should find the carrot juice aversive and decrease their bite rate relative to their bite rate prior to conditioning. This assay included a series of phases: acclimation (i.e. habituation to the experimental arena), baseline bite rate, contingent training (the aversive stimulus was paired with carrot juice), and a memory test (Supplementary Fig. 4). Snails were deprived of food for 24 h prior to these phases.

- Acclimation: Snails were first habituated to the testing area; they were placed individually in 60-mm-diameter petri dishes with 18 ml of UV-sterilized oxygenated artificial pond water and allowed to acclimate for 10 min.
- Baseline bite rate: 1 ml of UV-sterilized pond water was added to the petri dish and snails were left for 2 min, followed by another 1 ml of UV-sterilized pond water for another 2 min. The bite rate in pond water alone was used to account for the baseline biting behaviour of snails in the absence of a food stimulus as snails perform occasional biting behaviour that can vary by individual. The snails were returned to their aquaria for 1 h.
- Contingent training: Snails were placed on the petri dish and acclimated for 10 min. One millilitre of a 70% carrot juice solution was added (James White Organic Carrot Juice, James White Drinks, UK diluted with sterilized pond water), and the bite rate was recorded for 2 min (pretraining baseline bite rate in response to carrot juice), followed immediately by adding 1 ml of an aversive, unconditioned stimulus: KCl at 14.9 g/L. All snails stopped their bite response in response to the KCl stimulus. Snails were returned to their aquaria after a 2-min exposure to KCl.
- Memory test: Following a 24-h retention interval, snails were placed individually on a petri dish in 18 ml UV-sterilized pond water, with a 10-min acclimation, followed by 1 ml of pond water (where bite rate was recorded for 2 min), followed by 1 ml of 70% carrot juice solution, and the bite rate was recorded for a further 2 min.

To determine the response to training, the test bite rate was calculated as the difference between the bite rate in carrot compared to the bite rate in pond water to adjust for an increase in sporadic feeding behaviour in the absence of a food stimulus due to longer food deprivation. Memory was quantified as the change in bite rate: (bite rate in carrot juice during test adjusted for bite rate in pond water) − (bite rate in carrot juice during pretraining). These values were multiplied by −1 for analyses and graphical purposes, meaning higher values represent better memory.

We confirmed a change in bite response to carrot juice following contingent training is due to associative memory formation between carrot and the aversive stimulus KCl, rather than a change in bite response due to repeated exposure to carrot or handling during the experimental procedure, by carrying out noncontingent controls (in which the aversive stimulus [KCl] was not congruent with exposure to the carrot juice). These tests were performed with an additional 20 individual snails that were not included in the antibiotic treatments (described in Supplementary methods, Supplementary Fig. 4).

### Thigmotaxis and speed

Snails were returned to their aquarium for 1 day with access to food *ad libitum* before the thigmotaxis and speed assays. Snails were placed individually in the centre of 140-mm-diameter glass petri dishes with 90 ml of UV-sterilized oxygenated artificial pond water. Once snails had fully emerged (eyes and tentacles visible), their path was tracked for 15 min. Thigmotaxis was calculated as the proportion of time spent in contact with the edge of the experimental arena and speed as total distance travelled over 15 min. The assay for speed does not account for time spent not moving and could alternatively be described as distance covered. For consistency in terminology across studies, we use “speed” to describe this behaviour.

### Metabolic rate

We measured the metabolic rate on the same day as measurements of thigmotaxis and speed following an established protocol for *L. stagnalis* [39, 40]. Snails were placed individually in 125-ml sealed flasks with UV-sterilized oxygenated artificial pond water. A small magnetic bar was constantly mixing the water inside the flask and a grid prevented contact between the snail and the bar. Respirometry measurements were taken using a fibre-optic oxygen meter (FireStingO2, Pyroscience) to quantify the amount of oxygen (μmol/L/min) where an increased rate of decline in oxygen indicated higher metabolic rate [39, 40]. Snails were allowed to acclimate to the respirometry chambers for 20 min, and the rate of oxygen consumption was then calculated over a 20-min period to determine the metabolic rate.

### Gut microbiota analyses

Immediately following measurements of activity and metabolic rate, individuals were anesthetized in a 5% ethanol solution and immersed in a euthanizing 70% ethanol solution. Entire snail guts were dissected under sterile conditions, and the guts were preserved at −80°C. Microbial DNA was extracted using the Qiagen PowerSoil Pro kit following manufacturer’s instructions. For each snail sample, the entire dissected gut (<0.1 g) was added to the kit, alongside two negative controls. Ninety-five snail samples were randomly chosen across treatment groups (control *n =* 21, low *n =* 21, medium *n =* 24, and high *n =* 29).

The V3–V4 variable region of the 16S rRNA gene was amplified from the DNA extracts using the 16S amplicon sequencing library protocol (Illumina) as described in [16]. In the current study, each PCR amplification contained 5 μl of DNA, 10 μl for each forward and reverse primers (1 μM), and 25-μl Kapa HiFi Hotstart ready mix (Roche, Ireland) to a final volume of 50 μl. Three negative controls were run in parallel to sequencing: two from the DNA extraction stage and one containing PCR water instead of DNA template at the amplification stage. Successful PCR products were confirmed visually (gel agarose and a UV light box) and quantitatively using the Qubit high sensitivity kit. DNA bands were not observed in the negative controls and Qubit readings were “too low.” All experimental samples were successful (*n =* 95). All PCR products, including negative controls, were cleaned using AMPure XP magnetic bead–based purification (Labplan, Dublin, Ireland). Samples were sequenced at the Teagasc Sequencing Centre on the MiSeq sequencing platform, using a 2 × 300 cycle kit, following standard Illumina sequencing protocols.

### Bioinformatics

Adapters and low-quality bases from sequence data were trimmed using trimmomatic v 0.38 [41] with the parameters: HEADCROP:6 LEADING:20 SLIDINGWINDOW:4:30 MINLEN:200. Vsearch v2.10.4 [42] was used to merge paired reads and collapse of identical sequences using default parameters. Operational taxonomic unit (OTU) clustering was at 97% identity, with pairwise % identity calculated as (matching columns)/(alignment length). Chimeras were removed using uchime. Taxonomy was assigned using the Ribosomal Database Project Classifier (RDP) [43] with 16S rRNA reference (RDP) training set version 19, with a confidence threshold of 80%. The negative controls had very low reads (DNA: 58 and 300 reads, PCR: 339 reads) and were not included in downstream analyses. No decontamination steps were performed, as this methodology comes with the risk of removing host-relevant microbes. Our approach should not systematically bias samples or our aim to compare host phenotypes with gut microbiota.

We used the well-established clustering pipeline of assigning sequence data to 97% OTU similarity. We were primarily interested in general compositional associations between the gut microbiome and host traits, rather than splitting OTUs into higher taxonomic resolution to detect extremely rare and low abundant community members provided by 100% amplicon sequence variants. Moreover, 97% OTUs, rather than ASVs, may reduce variation across sequences for improved reference sequence matching and Nearest Sequenced Taxon Index (NSTI) scores and therefore reliability of PICRUSt2 output [44].

The outputs from above (OTU table, taxonomic table) and the metadata (treatments, behaviours) were analysed using phyloseq [45] in R statistical Software [46]. Sequences identified as chloroplast were removed (no OTUs were classified as mitochondrial), as were samples with less than 1000 reads (*n =* 1), in accordance with visual inspection of a rarefaction curve, using the function rarecurve() in the package vegan [47]. Following these filtering steps, one sample from the high antibiotic treatment was dropped (sample S30_L001). A total of 1 159 694 reads (mean per sample = 12 337.17, min *=* 1961, max = 187 835) clustered into 5127 OTUs across 94 samples. Reads were not rarefied prior to alpha diversity calculation or relative abundance analyses [48, 49].

### Statistical analyses

We ran LMMs using lme4 [50], and *P* values were obtained using lmerTest [51] in R version 3.5.2 [46]. Unless otherwise stated, models were run with a Gaussian distribution and residuals were checked for normality and homogeneity of dispersion. For models that contained multiple fixed terms, we used the dredge function from the MuMIn package [52] and an information-theoretic approach in combination with model averaging [53]. We generated models from a global model from our GLMMs and retained models with an Akaike’s information criterion corrected for small sample sizes (AICc) within seven units of the top model [54]. We report the conditional averaged weighted parameter estimates across the retained models. All continuous variables were scaled. Aquarium was included as a random effect. Plots were generated using ggplot2 [55].

#### Behaviours

Speed and thigmotaxis have been described as discrete measures of exploration in a novel arena [23]. Yet because the two variables were highly correlated in the control group, conceivably they could be measuring the same behavioural phenotype. Therefore, we performed a Principal Component Analyses using prcomp(), but Eigen values were low (PC1 = 0.08 and PC2 = 0.03), meaning we could not ascribe a common factor explaining thigmotaxis and speed. Using the vif() function, we also did not detect collinearity between all phenotypic variables that could otherwise influence statistical models, and all variance inflation factor values were low and close to 1.

#### Covariation between phenotypes

Pairwise covariation between phenotypes was tested using Pearson’s correlation, alongside 2000 bootstrap iterations to calculate upper 95% and lower 5% confidence intervals. We report *P* values; however, we consider covariation between traits to be a genuine effect if confidence intervals do not overlap zero. Each comparison was split between treatment groups.

#### Statistical analysis: antibiotic treatments on behaviour

We performed four separate LMMs as described above to test whether antibiotic treatment affected (i) memory (bite rate change), (ii) thigmotaxis, (iii) speed, and (iv) metabolic rate. Antibiotic treatment was a four-level factor (control, low concentration, medium concentration, high concentration).

#### Statistical analysis: alpha diversity

Three alpha diversity metrics were calculated using the function estimate_richness() from the package phyloseq [45]: Shannon index (abundance/richness and evenness), observed (richness), and Chao1 (richness accounting for rare taxa missed from under sampling). We tested whether alpha diversity was affected by antibiotic treatment and predicted by the three behavioural measures by performing three separate LMMs as described above. Our global models included the following fixed effects: treatment (four-level factor), bite range change, thigmotaxis, speed, and metabolic rate, and all their interactions. All interactions were nonsignificant, yet retained in the observed and Chao1 model, but not the Shannon index model. In case of model overfitting due to multiple interactions, we reran and report the observed and Chao1 models without interaction terms.

#### Statistical analysis: beta diversity

For beta diversity analyses, taxa present at <0.005% were removed following [56], and we applied cumulative sum scaling normalization to standardize library size across samples using the package metagenomeSeq [57]. We present data from two different distance metrics for beta diversity calculated in two ways: (i) Aitchinson’s distance matrix, which calculates the Euclidean distances between clr-transformed compositions according to shared OTUs and their abundances, and (ii) Jaccard distance matrix which does not account for differences in abundance. Each matrix was analysed using permutational multivariate analysis of variance (ADONIS) with 100 permutations. We tested for homogeneity of dispersion and found there were significant differences in dispersion between antibiotic treatment groups, (*F* = 5.4, *P =* .002) therefore significant PERMANOVA results for this variable could reflect differences in group variance rather than differences in group means, or could reflect differences in both group variance and group means [58].

Beta diversity was included as the response variable and treatment, bite rate change (X−1), thigmotaxis, speed, and metabolic rate were included as fixed terms. Models were fit with the argument “terms,” which analyses the effect of each term sequentially. We could not specify “strata” as aquarium due to function errors from the unbalanced blocking of these random terms.

#### Statistical analysis: functional analysis

We used PICRUSt2 [31, 59] to predict gut microbiota metagenome functions using default settings. This methodology generates a phylogenetic tree from 16S rRNA sequence data aligned to reference genomes to predict gene-family copy numbers for each OTU and produces an abundance table of KEGG Orthologue pathways which we used for downstream analyses. The output also provides MetaCyc pathways, but we opted to restrict our analyses to KEGG Orthologue pathways to limit multiple statistical tests, and because this allowed us to compare our inferred pathway results to a recent study identifying whole metagenome KEGG orthologues associated with memory in an invertebrate system [60]. 260 input sequences aligned poorly to reference sequences (--min_align optio*n =* 0.8) and were excluded. These input sequences were excluded from downstream steps. Otherwise, the weighted NSTI scores were very low (mean 0.04, +/− SE = 0.0046), indicating that microbes from our 16S rRNA sequence data were very closely related phylogenetically to fully described microbial genomes [31, 59].

#### Statistical analysis: differential abundance

We tested whether antibiotic treatment and the four phenotypes predicted the abundance of OTUs and inferred pathways using MaAsLin2 (Microbiome Multivariable Associations with Linear Models) with default settings. This differential abundance method best suited our data as it applies generalized LMMs, thus accommodating discrete variables (antibiotic treatment), multiple continuous variables (phenotypes), and random effects (aquarium) and ranked highly in a recent comparison of statistical differential abundance methods for 16S rRNA sequence data [61]. We report false discovery rate–corrected *P* values using the Benjamini–Hochberg method and discuss *P* < 0.1 as trends. We consider this approach to be conservative as false discovery rates are not necessarily applied in microbiome datasets [60, 62]. We performed two models (one for OTU abundance and one for KEGG ortholog [KO] pathway abundance) with antibiotic treatment, bite rate change (X −1), thigmotaxis, speed, and metabolic rate as fixed terms, and aquarium as a random term. MaASLin2 does not accommodate interaction terms.

## Results

### Antibiotic-induced gut microbiota perturbation is dose dependent

Pond snails were subjected to an acute antibiotic exposure at four different concentrations for 72 h: low (1 μg/L, phenotyped; sequenced sample size: *n =* 26; 21), medium (2 μg/L, *n =* 27; 24), high (4 μg/L, *n =* 32; 29), and a control pond water (*n =* 22; 21). Following treatments and phenotypic assays, we extracted microbial DNA from dissected whole guts. Here, we describe 16S rRNA sequence data of *L. stagnalis* gut microbiota (see also [63]). We characterized 5127 OTUs at 97% similarity across 12 classified phyla and 78 classified families (Supplementary Fig. 1; Supplementary Table 1).

**Figure 1:**
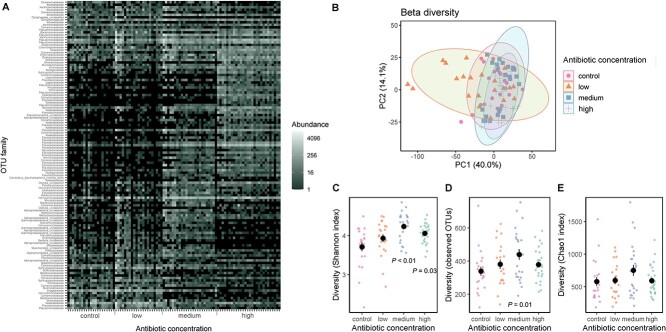
Gut microbiota metrics according to antibiotic treatment for (A) differentially abundant OTUs described at the family level or to the nearest classified level; (B) beta diversity calculated as Aitchinson’s distance; (C) Shannon index; and (D) observed species and (E) Chao1 diversity. Coloured dots represent individual samples; black dots represent mean and lines standard error. Statistics are detailed in Supplementary Table 1 (OTUs), Supplementary Table 2 (beta diversity), and Supplementary Table 4 (Shannon index, observed, and Chao1 diversity).

In line with our predictions, the gut microbiota was increasingly perturbed as antibiotic exposure increased across multiple metrics. Ten OTUs were differentially abundant at low concentration, 76 OTUs at medium concentration, and 117 OTUs at high concentration (Supplementary Table 1). Several OTUs decreased, but many also increased (Fig. 1A) due to the relative depletion of some highly abundant microbes and the colonization of novel microbes sourced from the environment. This interpretation reflects significant beta diversity (Supplementary Table 2) and clustering of community structure at medium and high concentrations away from low concentration and the control condition (Fig. 1B) for both Aitchinson distance, which considers relative abundance (*R*^2^ = 0.13, *P =* .001), and Jaccard distance, which is independent of abundance (Jaccard distances, *R*^2^ = 0.18, *P =* .001). The number of inferred KO functional pathways that were significantly differentially abundant increased with antibiotic concentration (14 KOs at low concentration, 659 at medium concentration, and 884 at high concentration, Supplementary Table 3).

The evenness (Shannon index) of the gut microbiota diversity increased at a threshold of 2 μg/L antibiotics and above (low: *z* = 1.23, *P =* .22; medium: *z* = 3.27; *P =* .001; high: *z* = 2.20, *P =* .03, Fig. 1C, Supplementary Table 4), which likely occurred because relatively highly abundant microbes were depleted. Antibiotic exposure increased observed richness and Chao1 diversity, an index of richness, at medium concentrations only (observed: low: *z* = 0.95, *P =* .34, medium: z = 2.43, *P =* .01, high: *z* = 0.89, *P =* .38, Fig 1D; Chao1: low: *z* = 0.10, *P =* .92; medium: *z* = 1.82, *P =* .07; high *z* = 0.13, *P =* .90, Fig. 1E, Supplementary Table 4), perhaps because depleted microbes opened niches to antibiotic resistant microbes. We also excluded the possibility that variation in the amount of carrot juice consumed by individual snails (as a potential dietary source of microbes) during the memory test explained the observed differences in alpha diversity (GLMM of total carrot juice consumed during training and test phases for Shannon index: *t*: 0.73, *P =* .20; observed: *t*: – 0.20, *P =* .83).

### Covariation between memory, behaviours, and metabolic rate

**Figure 2:**
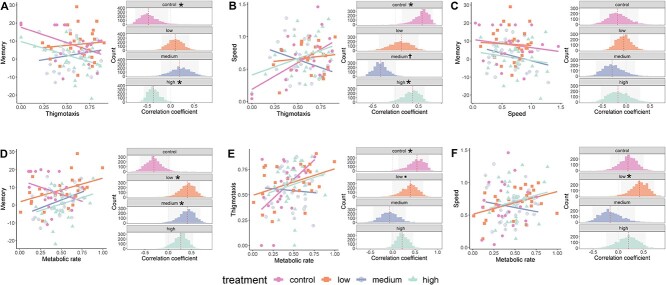
Correlations between phenotypic traits as individual-coloured dots and regression lines (left panel) and Pearson’s correlation coefficient across 2000 bootstrap iterations. (A) Thigmotaxis and memory, (B) thigmotaxis and speed, (C) speed and memory, (D) metabolic rate and memory, (E) metabolic rate and thigmotaxis, and (F) metabolic rate and speed. ^*^indicates correlated phenotypes where *P* < .05 and 95% CI do not overlap zero. † indicates correlated phenotypes where *P* < .1 and CI do not overlap zero. ▪ indicates correlated phenotypes where *P* < .1 and CI overlaps zero. Statistics are presented in Supplementary Table 5.

Snails were tested individually across four phenotypic assays following all treatments to test for covariation between traits and phenotypic plasticity. We performed a single-trial food conditioning paradigm [22, 64] to test memory formation of an aversive, unconditioned stimuli (potassium chloride, KCl) paired with an appetitive conditioned stimuli (carrot juice). Two assays for exploration measured the distance travelled over 15 min—speed—and the proportion of time spent in contact with the edge of a novel experimental arena—thigmotaxis. Routine metabolic rate was measured as the rate of oxygen consumption (μmol/L/min) following a 20-min acclimation period. Consistent with the pace-of-life theory, we found covariation (Supplementary Table 5), in the untreated control group, between speed and thigmotaxis (Pearson’s correlation test *P =* .004, *r* = .58, bootstrap confidence intervals (CI) from 2000 iterations = 0.14, 0.81, Fig. 2B), between thigmotaxis and metabolic rate (*P =* .01, *r* = .54, bootstrap CI = 0.12, 0.81, Fig. 2E), and between memory and thigmotaxis (*P =* .03, *r* = −.47, bootstrap CI = −0.71, −0.03, Fig. 2A). By contrast, the metabolic rate did not covary with speed (*P =* .39, *r* = .19, bootstrap CI = −0.16, 0.49, Fig. 2F) or memory (*P =* .16, *r* = −.31, bootstrap CI = −0.61, 0.05, Fig. 2D), and memory was not correlated with speed (*P =* .44, *r* = −.31, bootstrap CI = −0.54, 0.23, Fig. 2C).

### Antibiotic exposure leads to a decrease in memory and alters the covariation between pace-of-life traits

**Figure 3:**
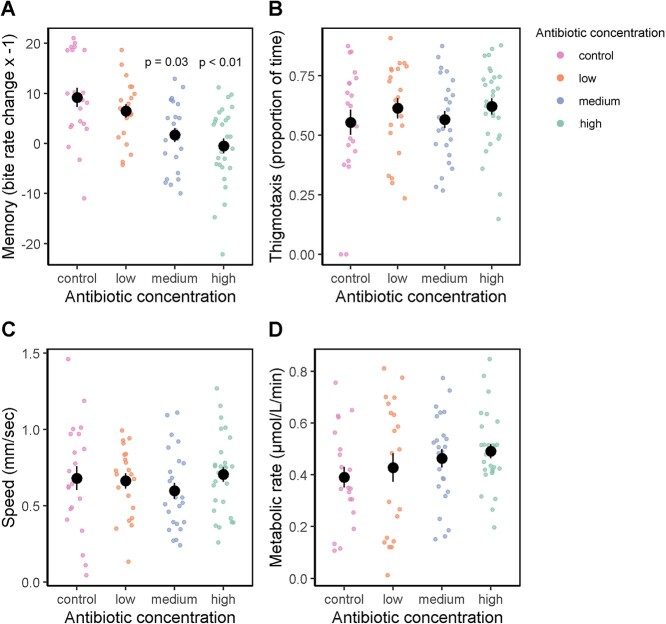
Effects of antibiotics on four phenotypes for (A) memory, (B) thigmotaxis, (C) speed, and (D) metabolic rate. Coloured dots represent individual samples; black dots represent mean and lines standard error. Statistics are presented in Supplementary Table 6.

We then tested whether the antibiotic treatments induced phenotypic plasticity and/or disrupted the covariation between traits. *Lymnaea stagnalis* has shown plasticity in memory [21, 39, 65], exploration behaviour [20, 39], and metabolic rate [39, 66] in response to environmental stressors including predation risk and social isolation, and we predicted that these phenotypes may also change in response to exposure to antibiotics and gut microbiome disruption. Snails had poorer memory formation at medium (*t* = −2.31, *P =* .03) and high (*t* = −3.04, *P* < .01) antibiotic concentrations, but not at low (*t* = −2.31, *P =* .27) (Fig. 3A), which reflect the antibiotic concentrations at which the gut microbiota was most perturbed. *Lymnaea stagnalis’* propensity to consume carrot juice in the pretraining trials did not differ from the control group (Supplementary Fig. 2) excluding the possibility that observed changes in bite rate were due to the gut microbiome’s influence on food palatability and preferences [67, 68].

Neither exploratory behaviours, nor metabolic rate differed across treatment groups (Fig. 3B–D, Supplementary Table 6). Instead, we show that antibiotic treatment disrupted the covariation (or lack of) between phenotypes, except for memory and speed (Fig. 2, Supplementary Table 6). Whether the covariation between traits was masked, revealed, or maintained was dependent on antibiotic dosage, and trait comparison. Regardless of the trait comparison, changes to covariation followed a nonlinear pattern across aquatic environments of increasing antibiotic concentration (Fig. 2, Supplementary Table 6).

### In line with gut microbiota perturbation, taxonomic and functional features of the gut microbiota explain individual variation in memory

**Figure 4:**
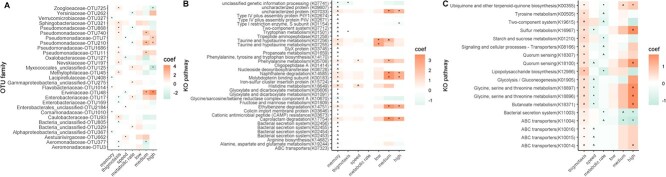
Differentially abundant gut microbiota features across phenotypic traits and antibiotic exposure. (A) Differentially abundant OTUs described at the family level. (B) Significantly differentially abundant KO pathways for memory and whether these KOs were or were not differentially abundant in pace-of-life traits (thigmotaxis, speed, and metabolic rate) and antibiotic treatments. (C) KO pathways that predict at least two pace-of-life traits and whether these KOs were perturbed by the antibiotic treatments. Coef indicates statistical correlation coefficient from MaASLin2 output. ^*^represents *P* < .05, ^ *P* < .01. *P* values are Benjamini–Hochberg-corrected for multiple comparisons. Full statistical outputs are presented in Supplementary Tables 7, 10, 11, and 14.

If the gut microbiota regulates host biology, individual variation in the gut microbiota should predict individual variation in phenotypes, and plasticity in phenotypes should reflect the antibiotic effect on the gut microbiota. We showed that phenotypic plasticity in memory formation was associated with five differentially abundant OTUs (Fig. 4A): OTU1014 (*Flavobacteriaceae*), OTU1010 (*Comamonadaceae*), OTU367 (unclassified *Alphaproteobacteria*), OTU408 (*Lacipirellulaceae*), and OTU327 (*Verrucomicrobiaceae*). OTU327 was also less abundant in snails exposed to the high antibiotic dose. Several KOs that predicted memory formation were also differentially abundant following antibiotic exposure, which we interpret as being the primary candidate functional pathways involved in microbiome-disrupted memory (Fig. 4B). These KOs match pathways involved in taurine and hypotaurine metabolism, phenylalanine metabolism, nephathalene degradation, ethylbenzene degradation, and caprolactam degradation. Several additional KO pathways predicted memory yet were not affected by antibiotic treatment. Of these pathways, tryptophan metabolism, two-component system, propanoate metabolism, histidine metabolism, glyoxylate and dicarboxylate metabolism, fructose and mannose metabolism, cationic antimicrobial peptide (CAMP) resistance system, and arginine biosynthesis have been reported in microbiome–gut–brain axis studies in other vertebrate and invertebrate systems (e.g. [1, 69–72]). We found no evidence that alpha diversity (Supplementary Fig. 3) nor beta diversity predicted memory, pointing to the inferred microbiome function as being the most important predictor for host cognition.

### Gut microbiota predicts exploration behaviours and metabolic rate, irrespective of gut microbiota perturbation

Several differentially abundant OTUs (Fig 4A, Supplementary Table 1) and hundreds of KO orthologs (Supplementary Table 3) predicted pace-of-life traits. There was weak, nonsignificant support for a positive association between thigmotaxis and Shannon index diversity (*z* = 1.87, *P =* .06, Supplementary Fig. 3B, Supplementary Table 4) and gut microbial community (Jaccard distance *R*^2^ = 0.01, *P =* .08, Supplementary Table 2). Alpha diversity did not explain individual variation for any other traits (Supplementary Fig. 2, Supplementary Table 4), and there was weak, nonsignificant evidence that community structure differed according to individual variation in speed (Jaccard distance: *R*^2^ = 0.01, *P =* .05, Supplementary Table 2).

### Candidate microbiota features may explain intercorrelation between phenotypes

The gut microbiome has been implicated as an intrinsic driver underlying intercorrelation between individual variation in pace-of-life phenotypes [15], perhaps because gut microbiome functions mediate suites of host traits uniformly. Alternatively, different gut microbiome features or functions may act independently on phenotypes, revealing or masking covariation [13] through homeostatic or allostatic processes [3]. Although we found no evidence for antibiotic-induced parallel changes across suites of host phenotypes, there was correlative evidence of microbiome features that predicted variation across more than one phenotype. Controlling for antibiotic exposure, OTU17 (*Enterobacteriaceae*), OTU868 (*Pseudomonadaceae*), and several inferred KO pathways predicted both thigmotaxis and the metabolic rate (ubiquinone biosynthesis, tyrosine metabolism, starch and sucrose metabolism, and others, Fig. 4B and C, Supplementary Table 7). Inferred KO pathways sulphur metabolism, glycolysis, butanoate metabolism, ABC transporters, and others predicted thigmotaxis and speed (Fig. 4C, Supplementary Table 7). There were no shared gut microbiota features that correlated with both speed and the metabolic rate (Supplementary Tables 8–11); therefore, none that were correlated across all three pace-of-life traits. Tryptophan metabolism predicted both memory and thigmotaxis, and histidine and phenylalanine metabolism predicted both memory and speed (Fig. 4B). Generally, microbiota features predominantly predicted variation in only one phenotype (Supplementary Tables 7–14).

## Discussion

Environmental stressors can reduce or block memory formation in many systems [73], including snails where cognitive sensitivity to stressors is population-specific [22, 65, 74, 75]. Our study is one of the few outside clinical studies to demonstrate the gut microbiota underlies observed environmental effects on cognitive plasticity [3]. We report no significant differences in behaviour or metabolic rate following antibiotic treatments, perhaps because these traits inherently have limited plasticity, yet individual trait values shifted sufficiently to cause a divergence in their covariation. Our findings also point to the gut microbiome as a promising framework for explaining why the pace-of-life literature is fraught with inconsistent reports [12]. We show that specific features of the gut microbiome may have pleiotropic effects on hosts due to their correlation with suites of traits, and we also show features that are uniquely associated with a single pace-of-life trait that are disrupted by antibiotic exposure and at levels that alter the intercorrelation with other phenotypes.

The absence of correlations between pace-of-life traits and nonconcurrent changes in correlated behaviours has been interpreted as evidence against pace-of-life theory [12]. Generally, our results support the idea that discrepancies stem from environmental stressors that mask or reveal pace-of-life syndromes by shifting within-population trait means and/or their variance [13]. Low and medium antibiotic concentrations were most likely to either mask or reveal covariation, and at high antibiotic concentrations, the presence or absence of covariation reflected results observed in the control condition. We speculate that this nonlinear pattern may reflect the magnitude of the stressor [13], where extreme stress triggers compensatory responses to maintain homeostasis [3, 76].

To what extent can we ascribe the gut microbiome as the mechanism mediating cognitive plasticity? We show that individual variation in cognition is associated with inferred KO pathways that match known microbiome–gut–brain axis mechanisms found in humans, rodents, and insects including tryptophan metabolism and phenylalanine metabolism (catecholamine neurotransmitters precursors) [71, 77, 78]; glyoxylate and dicarboxylate metabolism [79]; taurine metabolism [80]; and alanine, aspartate, and glutamate metabolism [70]. Many of the KO pathways listed here matched whole metagenomic KO pathways associated with memory in bumblebees (*Bombus terrestris*) [60]. At least three of these pathways/molecules are neuro-modulatory in *L. stagnalis* (tryptophan metabolism in the kynurenine pathway, glutamate, and taurine) [81–84]. Although our functional pathway analysis is limited in that it was inferred [31, 59], performance scores (weighted NSTI) indicated good sequence alignment to whole genome databases [31, 59]. We interpret these host biologically relevant functional associations, alongside parallel changes in the gut microbiome and memory in accordance with antibiotic concentrations, as support for microbiome–gut–brain axis processes operating in *L. stagnalis*.

Perhaps the most compelling inferred functional associations spanning pace-of-life traits was tyrosine metabolism, a precursor for catecholamines that are widely documented to be involved in locomotion and respiration in *L. stagnalis* ([85] and references therein). Microbial lipopolysaccharide biosynthesis activates gut cytokines involved in host immune responses [86], and the microbiome–gut–brain axis (reviewed in [87,88]). To our knowledge, only one other study investigated a suite of pace-of-life traits in the context of environmentally realistic alterations to the gut microbiota, suggesting temperature-adaptive developmental plasticity mediated by the gut microbiota [17]. We encourage more microbiome studies representing a wide taxonomic breadth to decipher whether these mechanisms are conserved or have evolved convergently.

It is conceivable that antibiotic effects on host traits occur independently from the gut microbiota by directly affecting the host through neuro-activity or toxicity [89]. Yet behavioural, physiological, and tissue-relevant gene transcription changes following antibiotic exposure [90–97] are widely interpreted to be mediated by host gut microbiomes [95, 98, 99]. Antibiotics may have acted as a cue for snails to adjust their feeding behaviour, although the snail’s general propensity to eat carrot juice in the pre-training trials (i.e. independent of memory) did not differ across antibiotic treatments. It is equally feasible that any imbalance to the gut microbiota ecosystem, irrespective of which gut microbiome features were perturbed, may explain deficits in memory [100]. The correlation between inferred functional pathways that have neuroactive potential in *L. stagnalis* potentially offers the strongest evidence that the gut microbiome may be mediating effects on memory. Future studies could implement targeted interventions of specific microbiota features identified here, particularly ones that have been genetically screened for relevant metabolic functions, and could aim to enhance, not just inhibit, cognition, although experimentally engineering complex gut microbial ecosystems remains logistically challenging [3, 101]. Because snails engage in coprophagy [[102], pers. obs], microbiome transplants to recapitulate phenotypes from one host to another could be one such approach provided the gut microbiota from donors successfully colonize those of the recipient [19]. Isolated microbial strain administration has also proven to be an effective tool for identifying the gut microbiome’s role in memory enhancement in bees [60].

From an ecological and conservation perspective, the disruptive effects of antibiotic pollution on wildlife are well documented [90–93]. Despite the numerous reports that antibiotics alter wildlife gut microbiomes [28], alongside clinical mechanistic evidence of the antibiotic-perturbed gut microbiota’s role in shaping host biology [91, 95–97], we have surprisingly little understanding of how antibiotic exposure in nature affects aquatic and soil organisms through their microbiomes (but see [103,104]). We make significant advances in this regard by demonstrating the most pronounced effects on the gut microbiota and memory occur at 2 μg/L and above, and disruption to covarying traits occurs at as little as 1 μg/L. Hundreds of OTUs and inferred KO pathways were altered due to antibiotic exposure, and they may have additional biological effects on the host not measured here. We found that antibiotic exposure increased gut microbiota alpha diversity, specifically by increasing taxonomic evenness, suggesting that many taxa were not killed by antibiotics, perhaps indicating a high occurrence of antibiotic-resistant genes in *L. stagnalis* [29]. Richness increased at medium antibiotic levels, presumably through exposure to environmental pools of microbes not measured here that were likely present in their aquarium, through coprophagy, or diet, although we show that the amount of carrot juice consumed did not explain between-treatment differences in alpha diversity. We can only speculate as to why the antibiotics had the strongest effects on alpha diversity at medium concentrations; it may be due to dose-dependent bacterial cellular responses to antibiotics [105].

Although we administered the antibiotic treatment as a combined cocktail, which is ecologically realistic [28–30], SMX and OTC differ in their antimicrobial actions and one antibiotic may have had more pronounced effects than the other. In wild snails, the gut microbiome and antibiotic exposure history may differ from the captive reared, F2 generation snails tested in our study, and consequently could affect microbiome and host phenotypic responses to antibiotic exposure. In nature, antibiotic exposure duration in the environment is highly variable [30], and our study investigated an acute antibiotic exposure of 72 h. Therefore, understanding differential effects and critical thresholds of a wide range of antibiotic pollutants, across different time scales, and organism life stages, will be valuable for environmental policy.

Predicting animal responses to unprecedented rates of environmental change are necessary if we are to mediate detrimental anthropogenic effects on biodiversity [106]. The gut microbiome may facilitate rapid phenotypic responses at a speed considerably faster than alternative host genetic adaptive mechanisms to match current environmental conditions [107], as shown for cold tolerance [108] and hibernation [109]. Equally, we predict that disruption to the gut microbiome may be so extreme that it leads to deleterious effects away from host fitness optimums. Distinguishing between these two complementary hypotheses should be a focus for future microbiome research to identify whether environmental perturbation of the gut microbiome through chemical pollutants leads to mismatches between host biology and the environment.

### Experimental organism

*Lymnaea stagnalis* used in these experiments were F2 generation adults (spire height 25 ± 1 mm) originally sourced from the Sowy River population on the Somerset Levels, UK. Animals were reared under laboratory conditions in aquaria containing oxygenated artificial pond water with 80 mg/L Ca2+ [22] at 20 ± 1°C on a 14:10 light:dark regime. Snails were fed with lettuce supplemented with trout pellets *ad libitum*. All individuals were identified throughout the experiments using queen bee tags (E. H. Thorne Ltd, UK) glued to the shell with nontoxic Loctite 454 adhesive (Henkel, UK). Experiments took place from June to August 2018. This research was in accordance with the ASAB (Association for the Study of Animal Behaviour) Guidelines for the Treatment of Animals in Behavioural Research and Teaching [110].

## Supplementary Material

Supplementary_data_Tables_wrae078

rdp_classified_GD_taxTable_wrae078

otu_table_centroids_iddef0_400bp_wrae078

metadata_full_wrae078

Snail_microbiome_SupplementaryR3_wrae078

Davidson_2024_gut_microbiota_explain_memory_RMarkdown_wrae078

## Data Availability

Sequence data are deposited at the National Center for Biotechnology Information (NCBI) Sequence Read Archive BioProject ID PRJNA1078756. All metadata and R code has been deposited on git hub: https://github.com/DrGLDavidson/lymnaeastagnalis-microbiome.
