# Supplementary material for: Antibiotic-altered gut microbiota explain host memory plasticity and disrupt pace-of-life covariation for an aquatic snail
Source: ISME J. 2024 May 30;18(1):wrae078. doi: 10.1093/ismejo/wrae078 (PMC11136587; doi:10.1093/ismejo/wrae078)
Supplement: Snail_microbiome_SupplementaryR3_wrae078 [file snail_microbiome_supplementaryr3_wrae078.docx]

Supplementary

Title: **Antibiotic-altered gut microbiota explain host memory plasticity and disrupt pace-of-life covariation for an aquatic snail.**

Gabrielle Davidson, Ignacio Alvarez-Cienfuegos, Sarah Dalesman

Supplementary figures 1-3

Supplementary methods and results

**A**


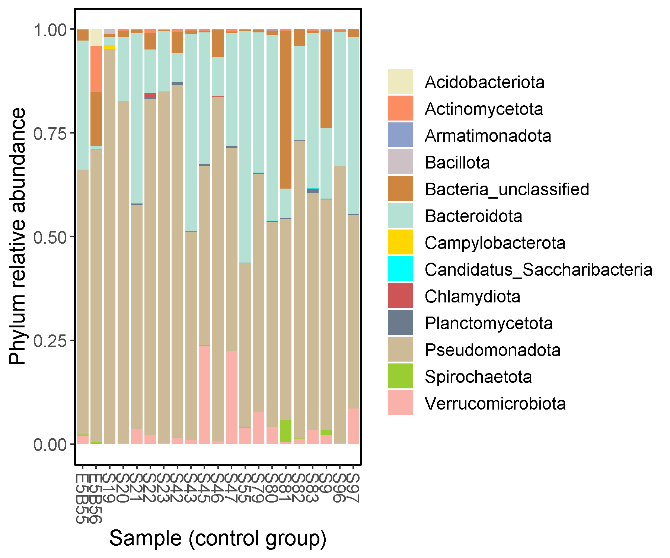


**B**


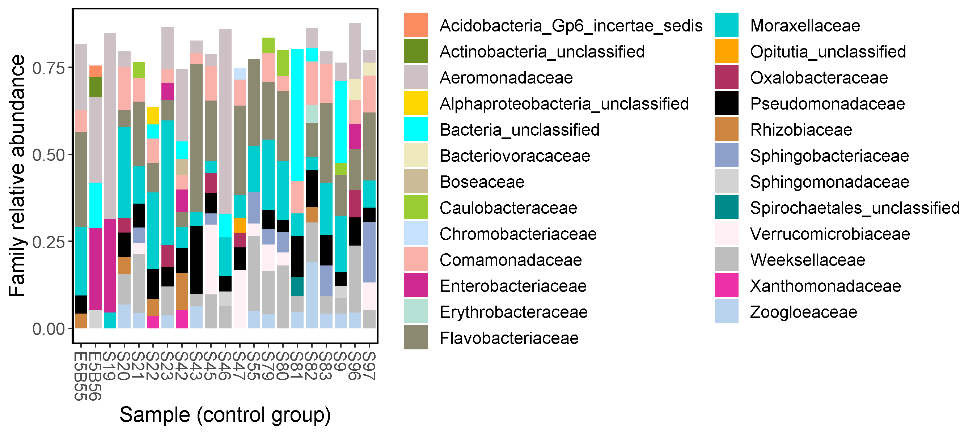


**C**

**
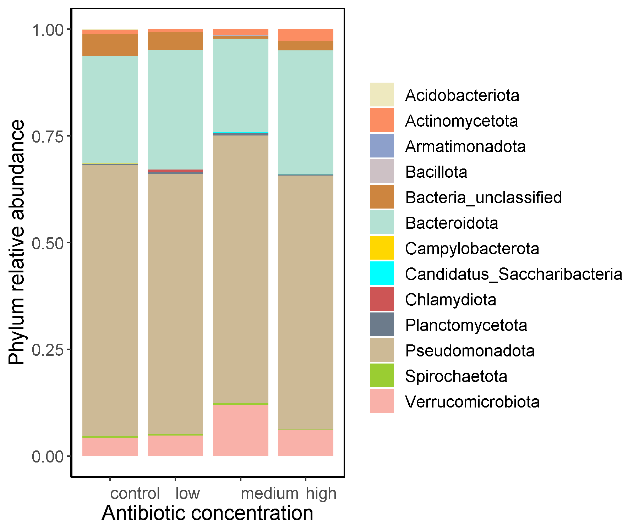
**

**D**


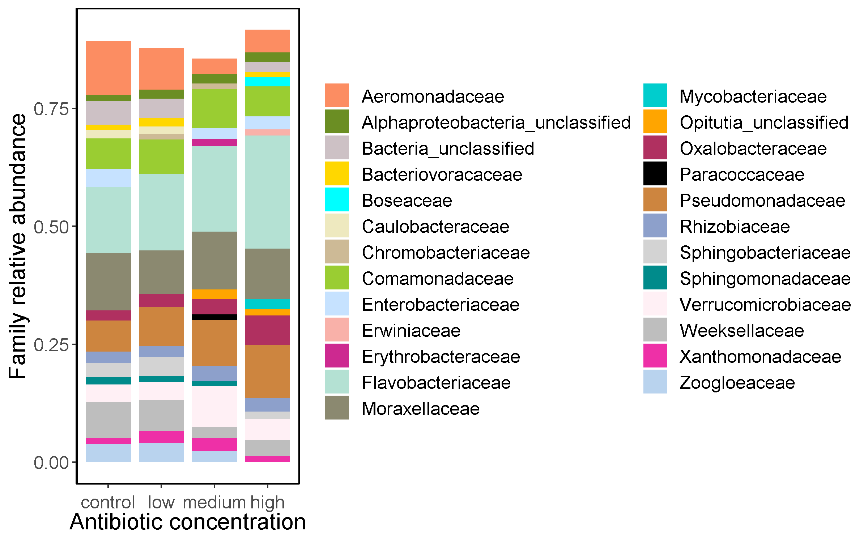


Supplementary Figure 1. Relative abundance of gut microbiota sequence reads per individual in the control groups for taxa present in >3% of samples. Family-level plots include the top abundant 25 families, and if not classified to the family-level, their nearest classification. **A)** The most abundant phylum-level taxa include *Pseudomonadota* (mean = 63.5%, +/- SE = 3.6), *Bacteroidota* (25.3%, +/-3.5), and *Verrucomicrobia* (4.3%, +/-1.5). B) The most abundant family-level taxa were *Flavobacteriaceae* (mean = 14.1%, +/- 2.4%), *Moraxellaceae* (12.1%, +/- 2.0), *Aeromonadaceae* (11.4%, +/- 3.4), *Weeksellaceae* (7.7, +/- 1.5), *Pseudomonadaceae* (6.5%, +/-0.9) and *Comamonadaceae* (6.4%, +/-0.8.). **B)** Relative abundance of gut microbiota sequence reads per treatment group for taxa present in >3% of samples. **C)** Irrespective of treatment, the most abundant phylum-level taxa include *Pseudomonadota* (mean = 69.2%, +/- SE = 1.0), *Bacteroidota* (26.2%, +/-1.3), *Verrucomicrobia* (6.9%, +/-0.6). **D)** Irrespective of treatment, the most abundant family-level taxa were *Flavobacteriaceae* (18.6%, +/- 1.0), *Moraxellaceae* (11.1%, +/-0.9), *Pseudomonadaceae* (9.2%, +/-0.6), *Verrucomicrobiaceae* (5.2%, +/-0.5), *Weeksellaceae* (4.8%, +/-0.5), Oxalobacteraceae (3.8%, +/-0.3).


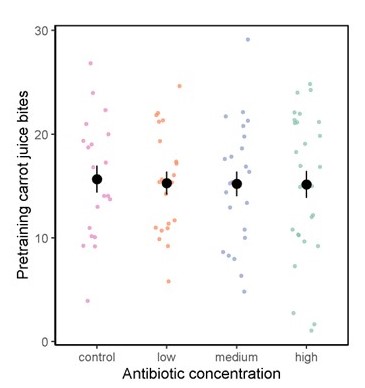


Supplementary Figure 2. Carrot juice bite rate during the pretraining trials, following antibiotic exposure but preceding exposure to aversive stimuli, showed know difference in the propensity to consume carrot juice across treatment groups, GLMM with control as the reference category: low: *t* = -0.308, *P* = 0.76; medium: *t* = -0.36, *P* = 0.72; high: *t* = -0.41, *P* = 0.68. This excludes the possibility that antibiotic treatment, and/or antibiotic-induced changes to the gut microbiome influenced carrot palatability.


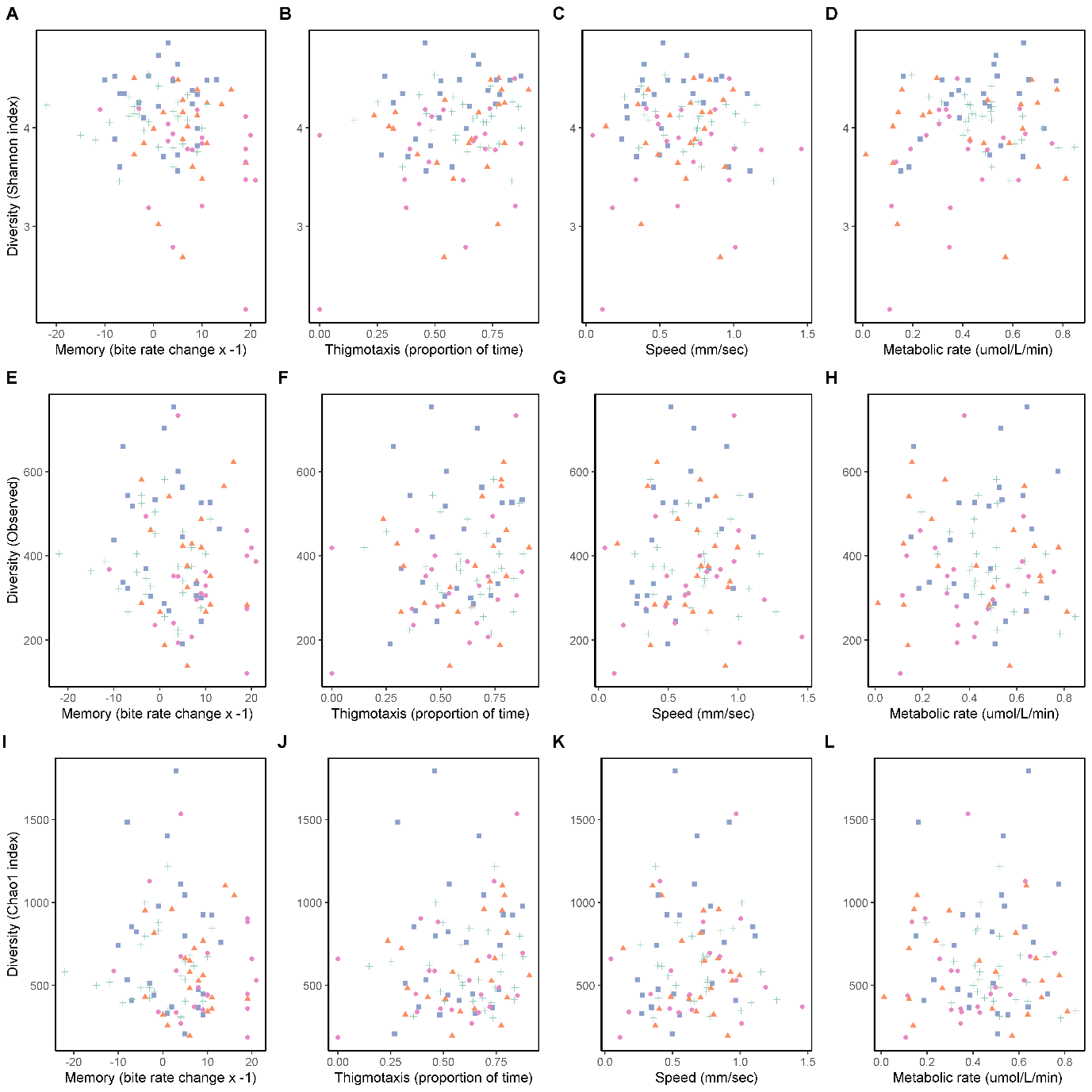


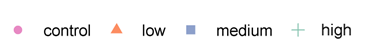


Supplementary Figure 3. Associations between Alpha diversity and phenotypic traits. Shannon diversity was weakly associated with thigmotaxis (B), but not memory (A), speed (C) or metabolic rate (D). Observed (E-H) and Chao1 (I-L) did not predict any phenotypic traits.

Supplementary Methods: Non-contingent controls

As with contingent training, snails were initially acclimated in 18 mL of UV sterlised pond water. Following 10 minutes, 1 mL of 70% carrot juice was added, and bite response recorded for 2 minutes. This was followed by the addition of 1 mL of pond water and snails were left for a further 2 minutes. An hour later, again following 10 minutes acclimation in 18 mL of pond water, 1 mL of pond water was added, followed 2 minutes later by 1 mL of KCl solution and snails were exposed for a further 2 minutes. This resulted in non-contingent snails experiencing the same handling and duration of exposure to each chemical, but exposure to carrot and KCl was separate by approximately 1 hour and 10 minutes (Figure 4). Linear Mixed Model within non-contingent controls confirmed that bite rate did not differ between pre and post training (*t* = 0.18, *P* = 0.86) (Supplementary Figure 5A). Linear models comparing contingent to non-contingent bite rate change confirmed that snails in contingent training had significantly higher bite rate change (mean = 8.48, SE = 1.9) their bite rate at test compared to non-contingent controls (mean bite rate change = 0.3, SE = 1.7)  *t* = -3.18, *P* = 0.003 (Supplementary Figure 5B).


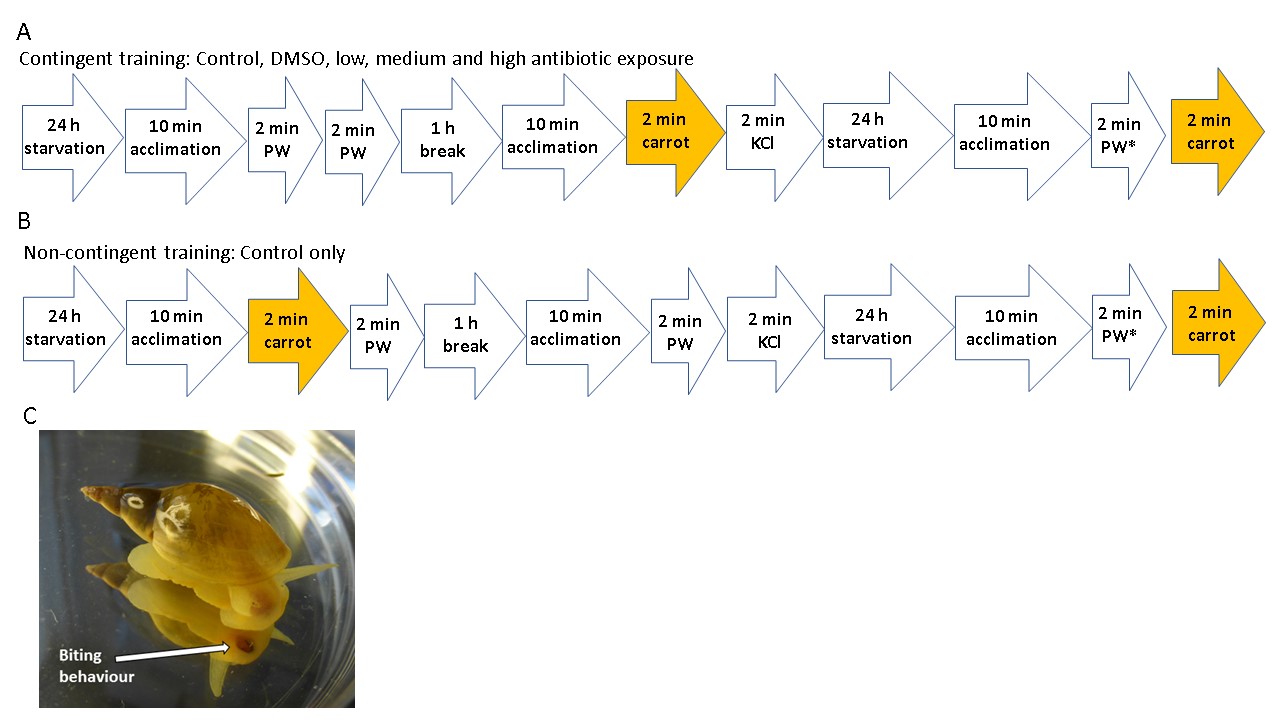


Supplementary Figure 4. Long term memory experimental procedure for A) contingent controls and B) non-contingent controls. C. Visible, open mouthpart showing biting behaviour.


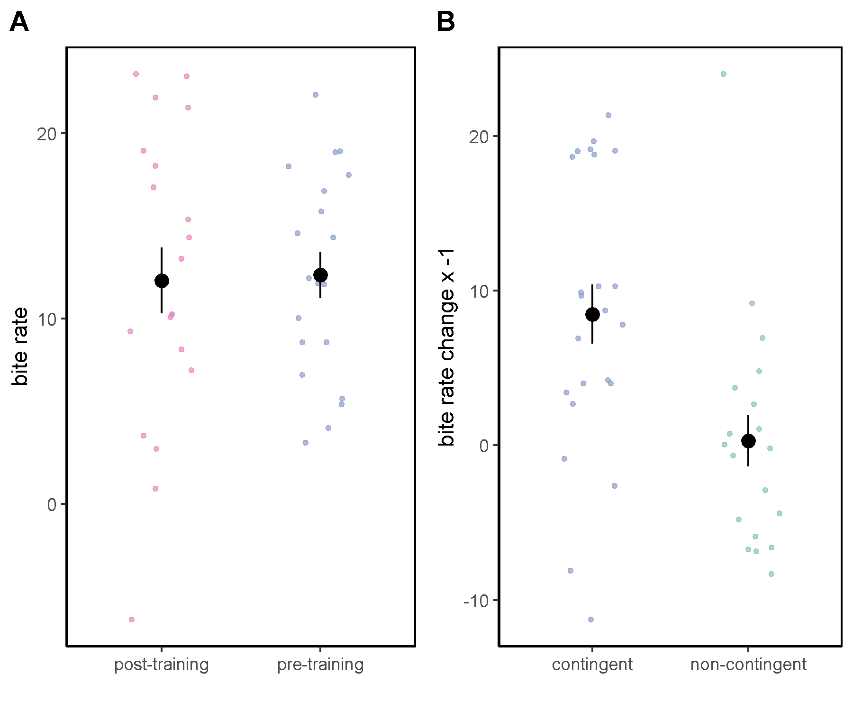


Supplementary Figure 5. A) bite rate within contingent controls for post training and pre training. B) bite rate change for non-contingent and contingent controls.
