# Supplementary material for: Antibiotic-altered gut microbiota explain host memory plasticity and disrupt pace-of-life covariation for an aquatic snail
Source: ISME J. 2024 May 30;18(1):wrae078. doi: 10.1093/ismejo/wrae078 (PMC11136587; doi:10.1093/ismejo/wrae078)
Supplement: Davidson_2024_gut_microbiota_explain_memory_RMarkdown_wrae078 [file davidson_2024_gut_microbiota_explain_memory_rmarkdown_wrae078.pdf]

Antibiotic-altered gut microbiota explain host memory plasticity and disrupt pace-of-life trait covariation for an aquatic snail

Gabrielle Davidson, Ignacio Alvarez-Cienfuegos, and Sarah Dalesman

2024-04-25

```
#load the following packages
library(vegan)
library(tidyverse)
library(phyloseq)
library(ggplot2)
library(dplyr)
library(lme4)
library(lmerTest)
library(metagenomeSeq)
setwd("F:/RWorkspace/Snail microbiome/snailMicrobiome Publication data")

taxonomy <- read.delim("rdp-classified_GD_taxTable.txt", header=TRUE)
metadata <- read.table("snail_metadata.txt", header=TRUE, row.names = "row.names") #before scal
ed data saved and alpha diversity
otus <- read.table("otu-table-centroids-iddef0-400bp.txt")

taxonomy <- data.frame(taxonomy[,-1], row.names=taxonomy[,1]) #set row.names

# Edit metadata for downstream analyses: invert the sign (-/+) for biteRateChange
# (more intuitive to interpret where higher values = better memory)


names(metadata)[names(metadata) == "biteRateChange"] <- "rawbiteRateChange"
metadata$biteRateChange = metadata$rawbiteRateChange*(-1)





##scale variables

metadata$scaledThig<-scale(metadata$Thigmotaxis)
metadata$scaledSpeed<-scale(metadata$Speed)
metadata$scaledBiteRate<-scale(metadata$biteRateChange)
metadata$scaledmetRate<-scale(metadata$metabolicRate)
names(metadata)

#phyloseq format
OTU = otu_table(otus, taxa_are_rows = TRUE)
TAX = tax_table(as.matrix(taxonomy))
MET = sample_data(metadata)

phyformat = phyloseq(OTU, TAX, MET)

# metadata with non-sequenced individuals and DMSO

#metadata<-read.csv("metadata_DMSO.csv",header=TRUE)
```

filtering steps

```
class(OTU) <- "matrix"
```

```
## Warning in class(OTU) <- "matrix": Setting class(x) to "matrix" sets attribute
## to NULL; result will no longer be an S4 object
```

```
tab <- t(OTU)
rarecurve(tab, step=50, cex=0.5, label = FALSE, xlim=c(0, 5000), ylim=c(0,600))
```

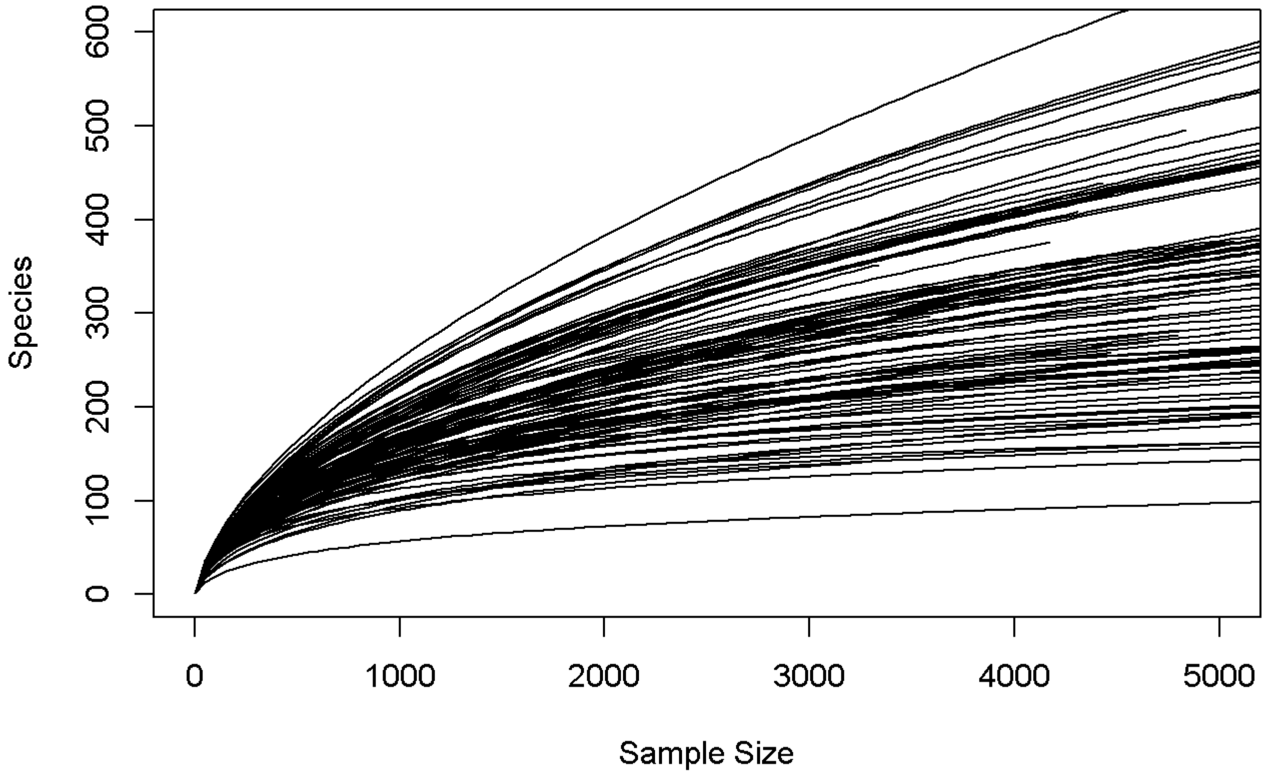

```
#a read cut off of 1000 is sufficient and conservative.

snailPhyloFilt <- prune_samples(sample_sums(phyformat)>=1000, phyformat)
# sample 30 has been dropped
# if using metadata object downstream, must remove sample 30

#remove cyanobacteria
filterPhyla = c("Cyanobacteriota")                #, "","","","")
snailPhyloFilt <- subset_taxa(snailPhyloFilt, !Phylum %in% filterPhyla)

#remove taxa w/ <0.005\% total abundance of total abundance of whole dataset recommeded by Boku
lich

# taxa total abundance
taxa.abundances <- get_sample(snailPhyloFilt)
# dataset total abundance
total.abundance <- apply(taxa.abundances, 1,sum)
# combine
tt.abundances <- cbind(taxa.abundances,total.abundance)
cutoff <- sum(total.abundance)*0.00005
keep <- total.abundance>cutoff
snailPhyloFilt2 <- prune_taxa(keep,snailPhyloFilt)

reads<-sample_sums(snailPhyloFilt2)
reads<-as.data.frame(reads)
sum(reads$reads)
```

```
## [1] 1159694
```

```
mean(reads$reads)
```

```
## [1] 12337.17
```

```
min(reads$reads)
```

```
## [1] 1961
```

```
max(reads$reads)
```

```
## [1] 187835
```

```
# NORMALISATION Transform OTU counts, using Cumulative sum scaling from metagenomeseq package.
# convert phylo obj to metagenomeSeq object
SnailMGS <- phyloseq_to_metagenomeSeq(snailPhyloFilt2)

# normalization
p <- cumNormStatFast(SnailMGS)
```

```
## Default value being used.
```

```
p
```

```
## [1] 0.5
```

```
SnailMGS <- cumNorm(SnailMGS, p =p)

#returns normalized factors for each sample
normFactors(SnailMGS)
```

```
##   S10_L001   S11_L001   S12_L001   S13_L001   S14_L001   S15_L001   S16_L001
##       1531       1428        845       1178        792        821        653
##   S17_L001   S18_L001   S19_L001    S1_L001   S20_L001   S21_L001   S22_L001
##        844        573        178        484        426        451        527
##   S23_L001   S25_L001   S26_L001   S27_L001   S28_L001   S29_L001    S2_L001
##        205        288        508        490        202        346        450
##   S31_L001   S32_L001   S33_L001   S34_L001   S35_L001   S36_L001   S37_L001
##        410        396        351        500        482        199        322
##   S38_L001   S39_L001    S3_L001   S40_L001   S41_L001   S42_L001   S43_L001
##        275        271        277        285        284        471        248
##   S44_L001   S45_L001   S46_L001   S47_L001   S48_L001   S49_L001    S4_L001
##        529        225        120        257        331        305        347
##   S50_L001   S51_L001   S52_L001   S53_L001   S54_L001   S55_L001   S56_L001
##        323        276        393        387        183        279        177
##   S57_L001   S58_L001   S59_L001    S5_L001   S61_L001   S62_L001   S63_L001
##        338        341        354        231        222        233        190
##   S64_L001   S65_L001   S66_L001   S67_L001   S68_L001   S69_L001    S6_L001
##        194        383        156        184        289        196        222
##   S70_L001   S71_L001   S72_L001   S73_L001   S74_L001   S75_L001   S76_L001
##        243         89        349        405        332        285        222
##   S77_L001   S78_L001   S79_L001    S7_L001   S80_L001   S81_L001   S82_L001
##        356        226        183        332        220        138         86
##   S83_L001   S85_L001   S86_L001   S87_L001   S88_L001   S89_L001    S8_L001
##        353        163        319        240        230        297        227
##   S90_L001   S92_L001   S93_L001   S94_L001   S95_L001   S96_L001   S97_L001
##        259        253        339        301        147        173        183
##    S9_L001 E5B55_L001 E5B56_L001
##        173        200        299
```

```
# To export normalized count matrix
CSS.count.matrix.snail <- MRcounts(SnailMGS, norm = T)

# reintegrate w/ phyloseq
CSS.otu.snail <- otu_table(CSS.count.matrix.snail, taxa_are_rows = TRUE)
snailPhyloCSS <- merge_phyloseq(CSS.otu.snail,snailPhyloFilt2)

# snailPhyloCSS is normalised for beta diversity analysis
# snailPhyloFilt and snailPhyloFilt2 is not normalised for alpha diversity analysis and relativ
e abundance analysis
```

preparing family and phylum for relative abundance

graphs

```
# collapse phyloseq object down to only contain Phyla level info ##
snailPhyloFilt2P <-  tax_glom(snailPhyloFilt2, "Phylum", NArm = TRUE)

## transform to relative abundances

snailPhyloRA <- transform_sample_counts(snailPhyloFilt2P, function(x){x / sum(x)})
# special phyloseq psmelt function retuns dataframe w/ otu abundances, taxa names and sample da
ta
motsnail <- psmelt(snailPhyloRA)
write.csv(motsnail, file = "otuAbundances_Taxa_Sample_Snail.csv")

phyloGlom = tax_glom(snailPhyloFilt2P, "Phylum")
glomTax = tax_table(phyloGlom)[,"Phylum"]
glomOTU = otu_table(phyloGlom)
glomTable = merge(glomOTU,glomTax,by=0,all=TRUE)
rownames(glomTable) = glomTable[,"Phylum"]
glomTable$Row.names = NULL
glomTable$Phylum = NULL

glomTable2 = glomTable / rep(colSums(glomTable), each = nrow(glomTable)) ###percentages
glomTable3 = as.data.frame(t(glomTable2))
write.csv(glomTable3, file = "RA_Phylum.csv")

SnailPhylumRA2023<-merge(glomTable3, metadata, by = 0)
write.csv(SnailPhylumRA2023, file = "metadata_PhylumRA.csv")
PhylumRA<-read.csv(file="metadata_PhylumRA.csv", header=TRUE)

# currently each column is a phylum with its abundance as values in cells. I want a column name
d Phylum and a column named Abundance
names(PhylumRA)
SnailPhylumRAGather<-gather(PhylumRA, 'Pseudomonadota','Verrucomicrobiota', 'Spirochaetota', 'B
acteria_unclassified' , 'Armatimonadota','Candidatus_Saccharibacteria',
 'Planctomycetota', 'Bacillota',  'Actinomycetota' ,  'Campylobacterota' ,'Bacteroidota', 'Acid
obacteriota',
 'Chlamydiota'  , key="Phylum", value = "Abundance")

snailMeans <- summarySE(SnailPhylumRAGather, measurevar="Abundance", groupvars=c("treatmentOrde
r","Phylum"))
write.csv(snailMeans, file="RAPhylumMeans.csv")
snailMeans2 <- summarySE(SnailPhylumRAGather, measurevar="Abundance", groupvars="Phylum")
write.csv(snailMeans2, file="RAPhylumMeansOverall.csv")
# same again but at Family level

# collapse phyloseq object down to only contain Phyla level info ##
snailPhyloFilt3 <-  tax_glom(snailPhyloFilt2, "Family", NArm = TRUE)

famGlom = tax_glom(snailPhyloFilt3, "Family")
glomTax = tax_table(famGlom)[,"Family"]
glomOTU = otu_table(famGlom)
glomTable = merge(glomOTU,glomTax,by=0,all=TRUE)

rownames(glomTable) = glomTable[,"Family"]
glomTable$Row.names = NULL
```

```
glomTable$family = NULL
glomTable<-subset(glomTable, select=-c(Family))
glomTable2 = glomTable / rep(colSums(glomTable), each = nrow(glomTable))
glomTable3 = as.data.frame(t(glomTable2))
write.csv(glomTable3, file = "RA_Family.csv")
SnailFamilyRA2023<-merge(glomTable3, metadata, by = 0)
write.csv(SnailFamilyRA2023, file = "metadata_FamilyRA.csv")

family<-rownames(glomTable2)
uniqueFamily<-unique(family)
SnailFamilyRAGather<-gather(SnailFamilyRA2023, uniqueFamily  , key="Family", value = "Abundanc
e")

snailMeans <- summarySE(SnailFamilyRAGather, measurevar="Abundance", groupvars=c("treatmentOrde
r","Family"))
write.csv(snailMeans, file="RAFamilyMeans.csv")
snailMeans2 <- summarySE(SnailFamilyRAGather, measurevar="Abundance", groupvars="Family")
write.csv(snailMeans2, file="RAFamilyMeansOverall.csv")
```

Beta Diversity

```
library(compositions)
```

```
# calc aitchison distance

clr.asvTable <- compositions::clr(snailPhyloCSS@otu_table)

#transform so columns are rows and vice versa
clr<-as.data.frame(clr.asvTable)
clr = t(clr)

aitchison.distances <- vegan::vegdist(clr, method = "euclid", na.rm=TRUE)
AD<-as.matrix(aitchison.distances)

# PERMUTATION TEST TO SEE IF VARIANCES DIFFER BY GROUPS
ADTreat<-vegan::betadisper(aitchison.distances, snailPhyloCSS@sam_data$treatmentOrder)
permutest(ADTreat, pairwise=FALSE, permutations=1000)
```

```
##
## Permutation test for homogeneity of multivariate dispersions
## Permutation: free
## Number of permutations: 1000
##
## Response: Distances
##           Df Sum Sq Mean Sq      F N.Perm   Pr(>F)
## Groups     3 162.39  54.130 5.4192   1000 0.003996 **
## Residuals 90 898.97   9.989
## ---
## Signif. codes:  0 '***' 0.001 '**' 0.01 '*' 0.05 '.' 0.1 ' ' 1
```

```
# Aitchinson Adonis by scaled continuous terms.

adonis2(aitchison.distances~snailPhyloCSS@sam_data$treatmentOrder+snailPhyloCSS@sam_data$scaled
BiteRate+snailPhyloCSS@sam_data$scaledSpeed + snailPhyloCSS@sam_data$scaledThig+snailPhyloCSS@s
am_data$scaledmetRate, method = "euclidian", by= "terms")
```

```
## Permutation test for adonis under reduced model
## Terms added sequentially (first to last)
## Permutation: free
## Number of permutations: 999
##
## adonis2(formula = aitchison.distances ~ snailPhyloCSS@sam_data$treatmentOrder + snailPhyloCS
S@sam_data$scaledBiteRate + snailPhyloCSS@sam_data$scaledSpeed + snailPhyloCSS@sam_data$scaledT
hig + snailPhyloCSS@sam_data$scaledmetRate, method = "euclidian", by = "terms")
##                                        Df SumOfSqs      R2      F Pr(>F)
## snailPhyloCSS@sam_data$treatmentOrder  3     4628 0.13418 4.6671  0.001 ***
## snailPhyloCSS@sam_data$scaledBiteRate  1      325 0.00943 0.9842  0.458
## snailPhyloCSS@sam_data$scaledSpeed     1      295 0.00856 0.8934  0.656
## snailPhyloCSS@sam_data$scaledThig      1      412 0.01196 1.2478  0.112
## snailPhyloCSS@sam_data$scaledmetRate   1      403 0.01168 1.2186  0.130
## Residual                              86    28425 0.82419
## Total                                 93    34488 1.00000
## ---
## Signif. codes:  0 '***' 0.001 '**' 0.01 '*' 0.05 '.' 0.1 ' ' 1
```

```
#jaccard distance

 otu_table<-snailPhyloCSS@otu_table

otu_table = t(otu_table)
 jaccard <- vegdist(otu_table, method = "jaccard", binary = TRUE)



# PERMUTATION TEST TO SEE IF VARIANCES DIFFER BY GROUPS
JTreat<-vegan::betadisper(jaccard, snailPhyloCSS@sam_data$treatmentOrder)
permutest(JTreat, pairwise=FALSE, permutations=1000)
```

```
##
## Permutation test for homogeneity of multivariate dispersions
## Permutation: free
## Number of permutations: 1000
##
## Response: Distances
##           Df  Sum Sq  Mean Sq      F N.Perm   Pr(>F)
## Groups     3 0.15856 0.052852 9.5348   1000 0.000999 ***
## Residuals 90 0.49888 0.005543
## ---
## Signif. codes:  0 '***' 0.001 '**' 0.01 '*' 0.05 '.' 0.1 ' ' 1
```

```
adonis2(jaccard~snailPhyloCSS@sam_data$treatmentOrder+snailPhyloCSS@sam_data$scaledBiteRate+sna
ilPhyloCSS@sam_data$scaledSpeed + snailPhyloCSS@sam_data$scaledThig+snailPhyloCSS@sam_data$scal
edmetRate, method = "jaccard", by= "terms")
```

```
## Permutation test for adonis under reduced model
## Terms added sequentially (first to last)
## Permutation: free
## Number of permutations: 999
##
## adonis2(formula = jaccard ~ snailPhyloCSS@sam_data$treatmentOrder + snailPhyloCSS@sam_data$s
caledBiteRate + snailPhyloCSS@sam_data$scaledSpeed + snailPhyloCSS@sam_data$scaledThig + snailP
hyloCSS@sam_data$scaledmetRate, method = "jaccard", by = "terms")
##                                       Df SumOfSqs      R2      F Pr(>F)
## snailPhyloCSS@sam_data$treatmentOrder  3   2.8983 0.18423 6.8760  0.001 ***
## snailPhyloCSS@sam_data$scaledBiteRate  1   0.1449 0.00921 1.0313  0.360
## snailPhyloCSS@sam_data$scaledSpeed     1   0.2184 0.01388 1.5542  0.044 *
## snailPhyloCSS@sam_data$scaledThig      1   0.1938 0.01232 1.3792  0.093 .
## snailPhyloCSS@sam_data$scaledmetRate   1   0.1934 0.01229 1.3762  0.100 .
## Residual                              86  12.0832 0.76807
## Total                                 93  15.7319 1.00000
## ---
## Signif. codes:  0 '***' 0.001 '**' 0.01 '*' 0.05 '.' 0.1 ' ' 1
```

Alpha diversity

```
alphaDiversitySnails<-estimate_richness(snailPhyloFilt, split = TRUE, measures = NULL)

alphaDiversitySnails<-alphaDiversitySnails%>%
  select(Chao1, Shannon, Observed)

# add a-div dataframe to metadata df
metadataAlphaDiversity <- merge(metadata, alphaDiversitySnails, by="row.names")

names(metadataAlphaDiversity)
```

```
##  [1] "Row.names"         "treatment"         "treatmentOrder"
##  [4] "aquarium"          "extractionRound"   "Thigmotaxis"
##  [7] "Speed"             "metabolicRate"     "Pretraining"
## [10] "Posttraining"      "rawbiteRateChange" "biteRateChange"
## [13] "scaledThig"        "scaledSpeed"       "scaledBiteRate"
## [16] "scaledmetRate"     "Chao1"             "Shannon"
## [19] "Observed"
```

```
write.table(metadataAlphaDiversity, file = "metadata_alphaDiversity.txt",sep="\t",row.names=FAL
SE)
```

clear environment and load packages and metadata for GLMM and pearsons corr tests

```
rm(list = ls(all.names = TRUE))
library(usdm)
library(MuMIn)
metadataFull<-read.csv(file="metadata_full.csv",header=TRUE)
```

Principal component and colinearity tests

```
#Speed and thigmotaxis are measures of exploration  in the same test - candidate for principle
component of these two traits?

names(metadataFull)[names(metadataFull) == "biteRateChange"] <- "rawbiteRateChange"
metadataFull$biteRateChange = metadataFull$rawbiteRateChange*(-1)

##scale variables

metadataFull$scaledThig<-scale(metadataFull$Thigmotaxis)
metadataFull$scaledSpeed<-scale(metadataFull$Speed)
metadataFull$scaledBiteRate<-scale(metadataFull$biteRateChange)
metadataFull$scaledmetRate<-scale(metadataFull$metabolicRate)
names(metadataFull)
```

```
##  [1] "uniqueID"          "Thigmotaxis"       "Speed"
##  [4] "Pretraining"       "Posttraining"      "rawbiteRateChange"
##  [7] "treatment"         "treatmentOrder"    "aquarium"
## [10] "metabolicRate"     "biteRateChange"    "scaledThig"
## [13] "scaledSpeed"       "scaledBiteRate"    "scaledmetRate"
```

```
PCA<-data.frame((metadataFull$Thigmotaxis), (metadataFull$Speed))
pcaout <- prcomp(PCA, center = TRUE, scale = TRUE )

pcaout<-prcomp(PCA)

eigen<-pcaout$sdev^2
eigen
```

```
## [1] 0.08400490 0.03672284
```

```
#below 1 eigen values therefore no justification to analyse speed and thigmotaxis as a principl
e component

# check if variables are colinear before including all in GLMM:

df<-data.frame(metadataFull$scaledmetRate, metadataFull$scaledThig, metadataFull$scaledSpeed, m
etadataFull$scaledBiteRate)
vif(df)
```

```
##                      Variables      VIF
## 1  metadataFull.scaledmetRate 1.151929
## 2     metadataFull.scaledThig 1.158081
## 3    metadataFull.scaledSpeed 1.109845
## 4 metadataFull.scaledBiteRate 1.098563
```

```
# all around 1 therefore no colinearity, can include in same model

# clean up environment
rm(PCA, pcaout, df, eigen, control)
```

```
## Warning in rm(PCA, pcaout, df, eigen, control): object 'control' not found
```

Correlation tests with bootstrap

```
# credit to bootstrapping tutorial https://www.datawim.com/post/bootstrapping-correlation-coeff
icients-in-r/
library(dplyr)
library(tidyverse)
library(tidymodels)
library(rstatix)
library(ggpubr)

metadataFull<-read.csv(file="metadata_full.csv",header=TRUE)
names(metadataFull)[names(metadataFull) == "biteRateChange"] <- "rawbiteRateChange"
metadataFull$biteRateChange = metadataFull$rawbiteRateChange*(-1)

##thigmotaxis metabolic rate

point <- metadataFull %>%
  group_by(treatmentOrder) %>%
  cor_test(Thigmotaxis, metabolicRate, method = "pearson")

point

boot_corr <- metadataFull %>%
  nest(data = -c(treatmentOrder)) %>% # grouping the treatments
  mutate(boots = map(data, ~bootstraps(.x, times = 2000, apparent = FALSE))) %>% # defining boo
tstraps
  unnest(boots) %>% # un-nesting bootsrapped data lists
  mutate(correlations = map(splits, ~cor_test(Thigmotaxis, metabolicRate, data = analysis
((.))))) # performing correlation

corr <- boot_corr %>%
  unnest(correlations) %>% # unnesting tidied data frames
  select(-data, -splits, -id)

corr

#confidence intervals from bootstrap
CI <- corr %>%
  group_by(treatmentOrder) %>%
        summarise(lwr_CI = quantile(cor, 0.025),
                  .estimate = median(cor),
                  upr_CI = quantile(cor, 0.975))

##speed metabolic rate

point1 <- metadataFull %>%
  group_by(treatmentOrder) %>%
  cor_test(metabolicRate, Speed, method = "pearson")

point1

boot_corr <- metadataFull %>%
  nest(data = -c(treatmentOrder)) %>% # grouping the treatments
  mutate(boots = map(data, ~bootstraps(.x, times = 2000, apparent = FALSE))) %>% # defining boo
tstraps
  unnest(boots) %>% # un-nesting bootsrapped data lists
  mutate(correlations = map(splits, ~cor_test(Speed, metabolicRate, data = analysis((.))))) # p
```

```
erforming correlation

corr <- boot_corr %>%
  unnest(correlations) %>% # unnesting tidied data frames
  select(-data, -splits, -id)

#confidence intervals from bootstrap
CI_1 <- corr %>%
  group_by(treatmentOrder) %>%
        summarise(lwr_CI = quantile(cor, 0.025),
                  .estimate = median(cor),
                  upr_CI = quantile(cor, 0.975))

##biteRate Thigmotaxis
point2 <- metadataFull %>%
  group_by(treatmentOrder) %>%
  cor_test(biteRateChange, Thigmotaxis, method = "pearson")

point2

boot_corr <- metadataFull %>%
  nest(data = -c(treatmentOrder)) %>% # grouping the treatments
  mutate(boots = map(data, ~bootstraps(.x, times = 2000, apparent = FALSE))) %>% # defining boo
tstraps
  unnest(boots) %>% # un-nesting bootsrapped data lists
  mutate(correlations = map(splits, ~cor_test(biteRateChange, Thigmotaxis, data = analysis
((.))))) # performing correlation

corr <- boot_corr %>%
  unnest(correlations) %>% # unnesting tidied data frames
  select(-data, -splits, -id)

#confidence intervals from bootstrap
CI_2 <- corr %>%
  group_by(treatmentOrder) %>%
        summarise(lwr_CI = quantile(cor, 0.025),
                  .estimate = median(cor),
                  upr_CI = quantile(cor, 0.975))

##biteRate Speed
point3 <- metadataFull %>%
  group_by(treatmentOrder) %>%
  cor_test(biteRateChange, Speed, method = "pearson")

point3

boot_corr <- metadataFull %>%
  nest(data = -c(treatmentOrder)) %>% # grouping the treatments
  mutate(boots = map(data, ~bootstraps(.x, times = 2000, apparent = FALSE))) %>% # defining boo
tstraps
  unnest(boots) %>% # un-nesting bootsrapped data lists
  mutate(correlations = map(splits, ~cor_test(biteRateChange, Speed, data = analysis((.))))) #
performing correlation

corr <- boot_corr %>%
  unnest(correlations) %>% # unnesting tidied data frames
```

```
  select(-data, -splits, -id)

#confidence intervals from bootstrap
CI_3 <- corr %>%
  group_by(treatmentOrder) %>%
        summarise(lwr_CI = quantile(cor, 0.025),
                  .estimate = median(cor),
                  upr_CI = quantile(cor, 0.975))

##biteRate metabolic rate
point4 <- metadataFull %>%
  group_by(treatmentOrder) %>%
  cor_test(biteRateChange, metabolicRate, method = "pearson")

point4

boot_corr <- metadataFull %>%
  nest(data = -c(treatmentOrder)) %>% # grouping the treatments
  mutate(boots = map(data, ~bootstraps(.x, times = 2000, apparent = FALSE))) %>% # defining boo
tstraps
  unnest(boots) %>% # un-nesting bootsrapped data lists
  mutate(correlations = map(splits, ~cor_test(biteRateChange, metabolicRate, data = analysis
((.))))) # performing correlation

corr <- boot_corr %>%
  unnest(correlations) %>% # unnesting tidied data frames
  select(-data, -splits, -id)

#confidence intervals from bootstrap
CI_4 <- corr %>%
  group_by(treatmentOrder) %>%
        summarise(lwr_CI = quantile(cor, 0.025),
                  .estimate = median(cor),
                  upr_CI = quantile(cor, 0.975))

##Thigmotaxis Speed
point5 <- metadataFull %>%
  group_by(treatmentOrder) %>%
  cor_test(Thigmotaxis, Speed, method = "pearson")

point5

boot_corr <- metadataFull %>%
  nest(data = -c(treatmentOrder)) %>% # grouping the treatments
  mutate(boots = map(data, ~bootstraps(.x, times = 2000, apparent = FALSE))) %>% # defining boo
tstraps
  unnest(boots) %>% # un-nesting bootsrapped data lists
  mutate(correlations = map(splits, ~cor_test(Thigmotaxis, Speed, data = analysis((.))))) # per
forming correlation

corr <- boot_corr %>%
  unnest(correlations) %>% # unnesting tidied data frames
  select(-data, -splits, -id)

#confidence intervals from bootstrap
CI_5 <- corr %>%
```

```
  group_by(treatmentOrder) %>%
        summarise(lwr_CI = quantile(cor, 0.025),
                  .estimate = median(cor),
                  upr_CI = quantile(cor, 0.975))


pearsonsResults<-bind_rows(point, point5, point1, point2, point3, point4)
write.csv(pearsonsResults, file = "pearsonsCorrelationResults.csv")
```

GLMMs

```
#treatments on behaviours



model4<-lmer(metabolicRate~ treatment + (1|aquarium), data = metadataFull)
summary(model4)
```

```
## Linear mixed model fit by REML. t-tests use Satterthwaite's method [
## lmerModLmerTest]
## Formula: metabolicRate ~ treatment + (1 | aquarium)
##    Data: metadataFull
##
## REML criterion at convergence: -42.2
##
## Scaled residuals:
##      Min       1Q   Median       3Q      Max
## -1.67373 -0.52076  0.00754  0.46166  2.49007
##
## Random effects:
##  Groups   Name        Variance Std.Dev.
##  aquarium (Intercept) 0.01756  0.1325
##  Residual             0.02236  0.1495
## Number of obs: 107, groups:  aquarium, 49
##
## Fixed effects:
##                 Estimate Std. Error       df t value Pr(>|t|)
## (Intercept)      0.39653    0.04810 68.97997   8.245 7.15e-12 ***
## treatmenthigh    0.10381    0.06346 66.30614   1.636    0.107
## treatmentlow     0.05787    0.06529 74.35471   0.886    0.378
## treatmentmedium  0.07914    0.06854 54.41979   1.155    0.253
## ---
## Signif. codes:  0 '***' 0.001 '**' 0.01 '*' 0.05 '.' 0.1 ' ' 1
##
## Correlation of Fixed Effects:
##             (Intr) trtmnth trtmntl
## treatmnthgh -0.728
## treatmentlw -0.678  0.493
## treatmntmdm -0.702  0.511   0.476
```

```
model6<-lmer(biteRateChange~treatment+(1|aquarium), data = metadataFull)
summary(model6)
```

```
## Linear mixed model fit by REML. t-tests use Satterthwaite's method [
## lmerModLmerTest]
## Formula: biteRateChange ~ treatment + (1 | aquarium)
##    Data: metadataFull
##
## REML criterion at convergence: 745.9
##
## Scaled residuals:
##      Min       1Q   Median       3Q      Max
## -2.67315 -0.57976  0.02409  0.70177  2.44678
##
## Random effects:
##  Groups   Name        Variance Std.Dev.
##  aquarium (Intercept)  1.008   1.004
##  Residual             71.048   8.429
## Number of obs: 107, groups:  aquarium, 49
##
## Fixed effects:
##                 Estimate Std. Error      df t value Pr(>|t|)
## (Intercept)       7.8986     1.8222 48.9878   4.335 7.24e-05 ***
## treatmenthigh    -7.2096     2.3684 45.2299  -3.044  0.00388 **
## treatmentlow      0.2523     2.4768 46.5907   0.102  0.91929
## treatmentmedium  -5.6741     2.4591 38.2166  -2.307  0.02654 *
## ---
## Signif. codes:  0 '***' 0.001 '**' 0.01 '*' 0.05 '.' 0.1 ' ' 1
##
## Correlation of Fixed Effects:
##             (Intr) trtmnth trtmntl
## treatmnthgh -0.769
## treatmentlw -0.735  0.565
## treatmntmdm -0.741  0.570   0.544
```

```
model7<-lmer(Thigmotaxis~treatment+(1|aquarium), data = metadataFull)
```

```
## boundary (singular) fit: see help('isSingular')
```

```
summary(model7)
```

```
## Linear mixed model fit by REML. t-tests use Satterthwaite's method [
## lmerModLmerTest]
## Formula: Thigmotaxis ~ treatment + (1 | aquarium)
##    Data: metadataFull
##
## REML criterion at convergence: -21.9
##
## Scaled residuals:
##      Min       1Q   Median       3Q      Max
## -2.96059 -0.54052  0.08709  0.74284  1.62195
##
## Random effects:
##  Groups   Name        Variance Std.Dev.
##  aquarium (Intercept) 0.00000  0.0000
##  Residual             0.04166  0.2041
## Number of obs: 107, groups:  aquarium, 49
##
## Fixed effects:
##                  Estimate Std. Error        df t value Pr(>|t|)
## (Intercept)       0.56897    0.04352 103.00000  13.075   <2e-16 ***
## treatmenthigh     0.03533    0.05653 103.00000   0.625    0.533
## treatmentlow      0.04970    0.05913 103.00000   0.841    0.403
## treatmentmedium  -0.02538    0.05862 103.00000  -0.433    0.666
## ---
## Signif. codes:  0 '***' 0.001 '**' 0.01 '*' 0.05 '.' 0.1 ' ' 1
##
## Correlation of Fixed Effects:
##             (Intr) trtmnth trtmntl
## treatmnthgh -0.770
## treatmentlw -0.736  0.567
## treatmntmdm -0.742  0.571   0.546
## optimizer (nloptwrap) convergence code: 0 (OK)
## boundary (singular) fit: see help('isSingular')
```

```
model8<-lmer(Speed~treatment+(1|aquarium), data = metadataFull)
```

```
## boundary (singular) fit: see help('isSingular')
```

```
summary(model8)
```

```
## Linear mixed model fit by REML. t-tests use Satterthwaite's method [
## lmerModLmerTest]
## Formula: Speed ~ treatment + (1 | aquarium)
##    Data: metadataFull
##
## REML criterion at convergence: 45.9
##
## Scaled residuals:
##      Min       1Q   Median       3Q      Max
## -2.16107 -0.67061 -0.00073  0.63261  2.82908
##
## Random effects:
##  Groups   Name        Variance Std.Dev.
##  aquarium (Intercept) 0.00000  0.0000
##  Residual             0.08047  0.2837
## Number of obs: 107, groups:  aquarium, 49
##
## Fixed effects:
##                  Estimate Std. Error        df t value Pr(>|t|)
## (Intercept)       0.65747    0.06048 103.00000  10.871   <2e-16 ***
## treatmenthigh     0.05829    0.07856 103.00000   0.742    0.460
## treatmentlow      0.02056    0.08217 103.00000   0.250    0.803
## treatmentmedium  -0.03163    0.08147 103.00000  -0.388    0.699
## ---
## Signif. codes:  0 '***' 0.001 '**' 0.01 '*' 0.05 '.' 0.1 ' ' 1
##
## Correlation of Fixed Effects:
##             (Intr) trtmnth trtmntl
## treatmnthgh -0.770
## treatmentlw -0.736  0.567
## treatmntmdm -0.742  0.571   0.546
## optimizer (nloptwrap) convergence code: 0 (OK)
## boundary (singular) fit: see help('isSingular')
```

```
#alpha diversity Shannon and Chao1

alphaDiversitySnails <-read.table("metadata_alphaDiversity.txt", row.names="Row.names", header
= TRUE)

# shannon
model1<-lmer(Shannon~scaledSpeed*treatmentOrder + scaledThig*treatmentOrder + scaledmetRate*tre
atmentOrder+ scaledBiteRate*treatmentOrder +treatmentOrder+ (1|aquarium), data=alphaDiversitySn
ails, na.action=na.fail)
summary(model1)
```

```
## Linear mixed model fit by REML. t-tests use Satterthwaite's method [
## lmerModLmerTest]
## Formula:
## Shannon ~ scaledSpeed * treatmentOrder + scaledThig * treatmentOrder +
##     scaledmetRate * treatmentOrder + scaledBiteRate * treatmentOrder +
##     treatmentOrder + (1 | aquarium)
##    Data: alphaDiversitySnails
##
## REML criterion at convergence: 128.3
##
## Scaled residuals:
##     Min      1Q  Median      3Q     Max
## -2.7813 -0.3677  0.1397  0.4820  1.8566
##
## Random effects:
##  Groups   Name        Variance Std.Dev.
##  aquarium (Intercept) 0.04712  0.2171
##  Residual             0.11361  0.3371
## Number of obs: 94, groups:  aquarium, 46
##
## Fixed effects:
##                                        Estimate Std. Error        df t value
## (Intercept)                            3.861159   0.114882 51.991051  33.610
## scaledSpeed                            0.014103   0.087381 72.739137   0.161
## treatmentOrderlow                      0.034565   0.159318 47.289894   0.217
## treatmentOrdermedium                   0.356660   0.154331 44.927757   2.311
## treatmentOrderxHigh                    0.253167   0.156532 44.732861   1.617
## scaledThig                             0.040158   0.113689 72.160650   0.353
## scaledmetRate                          0.125011   0.109043 73.921213   1.146
## scaledBiteRate                        -0.098595   0.090266 72.847669  -1.092
## scaledSpeed:treatmentOrderlow          0.062353   0.148094 64.376065   0.421
## scaledSpeed:treatmentOrdermedium       0.051362   0.121960 65.819225   0.421
## scaledSpeed:treatmentOrderxHigh       -0.118113   0.125816 73.217396  -0.939
## treatmentOrderlow:scaledThig           0.036196   0.143218 73.216544   0.253
## treatmentOrdermedium:scaledThig        0.150332   0.150083 73.936446   1.002
## treatmentOrderxHigh:scaledThig        -0.009395   0.145759 73.846197  -0.064
## treatmentOrderlow:scaledmetRate       -0.198571   0.139511 72.793567  -1.423
## treatmentOrdermedium:scaledmetRate     0.058081   0.143865 72.873307   0.404
## treatmentOrderxHigh:scaledmetRate     -0.273563   0.160020 71.853472  -1.710
## treatmentOrderlow:scaledBiteRate       0.156899   0.146560 69.677525   1.071
## treatmentOrdermedium:scaledBiteRate -0.023406   0.143772 72.157449  -0.163
## treatmentOrderxHigh:scaledBiteRate     0.101478   0.129412 73.987235   0.784
##                                      Pr(>|t|)
## (Intercept)                            <2e-16 ***
## scaledSpeed                            0.8722
## treatmentOrderlow                      0.8292
## treatmentOrdermedium                   0.0255 *
## treatmentOrderxHigh                    0.1128
## scaledThig                             0.7249
## scaledmetRate                          0.2553
## scaledBiteRate                         0.2783
## scaledSpeed:treatmentOrderlow          0.6751
## scaledSpeed:treatmentOrdermedium       0.6750
## scaledSpeed:treatmentOrderxHigh        0.3509
## treatmentOrderlow:scaledThig           0.8012
```

```
## treatmentOrdermedium:scaledThig       0.3198
## treatmentOrderxHigh:scaledThig        0.9488
## treatmentOrderlow:scaledmetRate       0.1589
## treatmentOrdermedium:scaledmetRate    0.6876
## treatmentOrderxHigh:scaledmetRate     0.0917 .
## treatmentOrderlow:scaledBiteRate      0.2881
## treatmentOrdermedium:scaledBiteRate   0.8711
## treatmentOrderxHigh:scaledBiteRate    0.4355
## ---
## Signif. codes:  0 '***' 0.001 '**' 0.01 '*' 0.05 '.' 0.1 ' ' 1
```

```
##
## Correlation matrix not shown by default, as p = 20 > 12.
## Use print(x, correlation=TRUE)  or
##     vcov(x)        if you need it
```

```
dd<-dredge(model1, subset= ~treatmentOrder, evaluate=TRUE, rank=AICc)
```

```
## Warning in dredge(model1, subset = ~treatmentOrder, evaluate = TRUE, rank =
## AICc): comparing models fitted by REML
```

```
## Fixed term is "(Intercept)"
```

```
dd
```

```
## Global model call: lmer(formula = Shannon ~ scaledSpeed * treatmentOrder + scaledThig *
##     treatmentOrder + scaledmetRate * treatmentOrder + scaledBiteRate *
##     treatmentOrder + treatmentOrder + (1 | aquarium), data = alphaDiversitySnails,
##     na.action = na.fail)
## ---
## Model selection table
##     (Int)      sBR     scR       scS     scT trO sBR:trO scR:trO scS:trO
## 17  3.755                                      +
## 25  3.760                            0.07368   +
## 19  3.773          0.04742                     +
## 18  3.774 -0.03086                             +
## 21  3.753                   0.015240           +
## 26  3.774 -0.02404                   0.07208   +
## 27  3.766          0.01933           0.06701   +
## 29  3.760                  -0.007105 0.07587   +
## 20  3.795 -0.03570 0.05055                     +
## 23  3.771          0.04582  0.009522           +
## 83  3.817          0.18390                     +               +
## 22  3.771 -0.02857          0.009464           +
## 281 3.766                            0.13780   +
## 28  3.784 -0.02714 0.02318           0.06393   +
## 30  3.777 -0.02692         -0.012420 0.07555   +
## 91  3.804          0.13450           0.07091   +               +
## 31  3.767          0.01950 -0.007846 0.06939   +
## 50  3.851 -0.14850                             +       +
## 24  3.794 -0.03539 0.05006  0.001703           +
## 149 3.746                   0.069630           +                       +
## 84  3.829 -0.02513 0.17700                     +               +
## 87  3.814          0.18060  0.011570           +               +
## 282 3.787 -0.03583                   0.12580   +
## 283 3.773          0.02059           0.12870   +
## 285 3.767                  -0.009555 0.14470   +
## 157 3.760                   0.021480 0.08079   +                       +
## 58  3.835 -0.11830                   0.06231   +       +
## 32  3.788 -0.03063 0.02420 -0.014160 0.06758   +
## 92  3.814 -0.01813 0.13070           0.06948   +               +
## 95  3.805          0.13500 -0.005435 0.07257   +               +
## 52  3.858 -0.14070 0.03568                     +       +
## 54  3.848 -0.14630          0.010900           +       +
## 151 3.763          0.04426  0.061550           +                       +
## 150 3.769 -0.03840          0.065880           +                       +
## 88  3.827 -0.02350 0.17550  0.006771           +               +
## 284 3.799 -0.04129 0.02831           0.11150   +
## 286 3.790 -0.03739         -0.013390 0.13490   +
## 287 3.774          0.02049 -0.009634 0.13580   +
## 158 3.781 -0.03520          0.018530 0.07976   +                       +
## 159 3.765          0.01680  0.021710 0.07525   +                       +
## 60  3.839 -0.11740 0.01458           0.05752   +       +
## 62  3.836 -0.11880         -0.007538 0.06469   +       +
## 96  3.816 -0.02043 0.13120 -0.009447 0.07194   +               +
## 152 3.793 -0.04488 0.04960  0.056270           +                       +
## 56  3.856 -0.13990 0.03450  0.005422           +       +
## 347 3.802          0.12370           0.08671   +               +
## 116 3.880 -0.11510 0.15240                     +       +       +
## 215 3.809          0.17210  0.041890           +               +       +
```

```
## 288 3.803 -0.04302 0.02855 -0.014150 0.12110   +
## 160 3.791 -0.03868 0.02292  0.018600 0.07220   +                       +
## 314 3.825 -0.09853                   0.10370   +       +
## 413 3.772                   -0.020410 0.15010   +                       +
## 64  3.840 -0.11790 0.01534 -0.008814 0.06009   +       +
## 124 3.853 -0.08828 0.10760           0.07558   +       +       +
## 223 3.807          0.12520 -0.002233 0.08451   +               +       +
## 348 3.823 -0.03645 0.12230           0.07462   +               +
## 182 3.837 -0.13800          0.052120           +       +               +
## 351 3.801          0.12390  0.001415 0.08583   +               +
## 120 3.877 -0.11360 0.14970  0.010790           +       +       +
## 216 3.827 -0.03286 0.16360  0.039660           +               +       +
## 414 3.796 -0.04265         -0.013260 0.13100   +                       +
## 316 3.833 -0.09810 0.02555           0.09270   +       +
## 415 3.778          0.01882 -0.017920 0.14030   +                       +
## 318 3.826 -0.09735         -0.007068 0.10930   +       +
## 190 3.831 -0.11240          0.015950 0.06732   +       +               +
## 224 3.822 -0.02775 0.11910 -0.003415 0.08309   +               +       +
## 128 3.853 -0.08868 0.10810 -0.006084 0.07738   +       +       +
## 184 3.846 -0.13140 0.03462  0.047150           +       +               +
## 352 3.823 -0.03675 0.12150 -0.002383 0.07677   +               +
## 416 3.807 -0.04911 0.02932 -0.008116 0.11280   +                       +
## 320 3.833 -0.09669 0.02584 -0.008290 0.09920   +       +
## 192 3.835 -0.11130 0.01432  0.016190 0.06301   +       +               +
## 380 3.858 -0.09606 0.12020           0.05463   +       +       +
## 479 3.806          0.12450 -0.001953 0.08558   +               +       +
## 248 3.871 -0.11000 0.14440  0.032570           +       +       +       +
## 446 3.828 -0.09794         -0.005017 0.10590   +       +               +
## 256 3.855 -0.08539 0.10410 -0.008733 0.08432   +       +       +       +
## 384 3.858 -0.09714 0.12190  0.006764 0.04899   +       +       +
## 480 3.830 -0.04340 0.12470  0.005244 0.06558   +               +       +
## 448 3.834 -0.09804 0.02548 -0.001296 0.09260   +       +               +
## 512 3.861 -0.09859 0.12500  0.014100 0.04016   +       +       +       +
##     scT:trO df  logLik  AICc delta weight
## 17           6 -49.802 112.6  0.00  0.746
## 25           7 -50.386 116.1  3.50  0.129
## 19           7 -51.441 118.2  5.61  0.045
## 18           7 -51.751 118.8  6.23  0.033
## 21           7 -52.019 119.3  6.77  0.025
## 26           8 -52.427 122.5  9.98  0.005
## 27           8 -52.461 122.6 10.05  0.005
## 29           8 -52.624 122.9 10.37  0.004
## 20           8 -53.311 124.3 11.75  0.002
## 23           8 -53.695 125.1 12.51  0.001
## 83          10 -51.271 125.2 12.62  0.001
## 22           8 -53.982 125.7 13.09  0.001
## 281       + 10 -52.646 127.9 15.37  0.000
## 28           9 -54.453 129.0 16.48  0.000
## 30           9 -54.612 129.4 16.80  0.000
## 91          11 -52.101 129.4 16.85  0.000
## 31           9 -54.693 129.5 16.96  0.000
## 50          10 -53.928 130.5 17.94  0.000
## 24           9 -55.556 131.3 18.68  0.000
## 149         10 -54.531 131.7 19.14  0.000
## 84          11 -53.297 131.8 19.24  0.000
## 87          11 -53.533 132.3 19.72  0.000
```

```
## 282       + 11 -54.491 134.2 21.63  0.000
## 283       + 11 -54.705 134.6 22.06  0.000
## 285       + 11 -54.807 134.8 22.26  0.000
## 157         11 -55.006 135.2 22.66  0.000
## 58          11 -55.105 135.4 22.86  0.000
## 32          10 -56.621 135.9 23.32  0.000
## 92          12 -54.199 136.3 23.68  0.000
## 95          12 -54.370 136.6 24.02  0.000
## 52          11 -55.796 136.8 24.24  0.000
## 54          11 -56.149 137.5 24.95  0.000
## 151         11 -56.243 137.7 25.13  0.000
## 150         11 -56.341 137.9 25.33  0.000
## 88          12 -55.554 139.0 26.39  0.000
## 284       + 12 -56.450 140.8 28.18  0.000
## 286       + 12 -56.623 141.1 28.53  0.000
## 287       + 12 -56.862 141.6 29.01  0.000
## 158         12 -56.873 141.6 29.03  0.000
## 159         12 -57.074 142.0 29.43  0.000
## 60          12 -57.188 142.2 29.66  0.000
## 62          12 -57.307 142.5 29.89  0.000
## 96          13 -56.423 143.4 30.83  0.000
## 152         12 -57.931 143.7 31.14  0.000
## 56          12 -58.026 143.9 31.33  0.000
## 347       + 14 -55.421 144.2 31.59  0.000
## 116         14 -55.831 145.0 32.41  0.000
## 215         14 -56.293 145.9 33.33  0.000
## 288       + 13 -58.574 147.7 35.13  0.000
## 160         13 -58.875 148.3 35.73  0.000
## 314       + 14 -57.526 148.4 35.80  0.000
## 413       + 14 -57.554 148.4 35.85  0.000
## 64          13 -59.374 149.3 36.73  0.000
## 124         15 -56.616 149.4 36.82  0.000
## 223         15 -56.710 149.6 37.00  0.000
## 348       + 15 -57.247 150.6 38.08  0.000
## 182         14 -58.688 150.7 38.12  0.000
## 351       + 15 -57.601 151.4 38.79  0.000
## 120         15 -58.061 152.3 39.70  0.000
## 216         15 -58.188 152.5 39.96  0.000
## 414       + 15 -59.275 154.7 42.13  0.000
## 316       + 15 -59.498 155.1 42.58  0.000
## 415       + 15 -59.591 155.3 42.77  0.000
## 318       + 15 -59.663 155.5 42.91  0.000
## 190         15 -59.781 155.7 43.15  0.000
## 224         16 -58.680 156.4 43.86  0.000
## 128         16 -58.845 156.8 44.19  0.000
## 184         15 -60.554 157.3 44.69  0.000
## 352       + 16 -59.417 157.9 45.33  0.000
## 416       + 16 -61.188 161.4 48.87  0.000
## 320       + 16 -61.625 162.3 49.75  0.000
## 192         16 -61.828 162.7 50.15  0.000
## 380       + 18 -59.539 164.2 51.63  0.000
## 479       + 18 -59.996 165.1 52.54  0.000
## 248         18 -60.851 166.8 54.25  0.000
## 446       + 18 -62.298 169.7 57.15  0.000
## 256         19 -61.435 171.1 58.57  0.000
## 384       + 19 -61.679 171.6 59.06  0.000
```

```
## 480       + 19 -61.697 171.7 59.09  0.000
## 448       + 19 -64.232 176.7 64.16  0.000
## 512       + 22 -64.143 186.5 73.97  0.000
## Models ranked by AICc(x)
## Random terms (all models):
##   1 | aquarium
```

```
ddAIC_S<-subset(dd, delta < 7)
ddAIC_S
```

```
## Global model call: lmer(formula = Shannon ~ scaledSpeed * treatmentOrder + scaledThig *
##     treatmentOrder + scaledmetRate * treatmentOrder + scaledBiteRate *
##     treatmentOrder + treatmentOrder + (1 | aquarium), data = alphaDiversitySnails,
##     na.action = na.fail)
## ---
## Model selection table
##    (Int)      sBR     scR     scS     scT trO df  logLik  AICc delta weight
## 17 3.755                                    +  6 -49.802 112.6  0.00  0.762
## 25 3.760                          0.07368   +  7 -50.386 116.1  3.50  0.132
## 19 3.773          0.04742                   +  7 -51.441 118.2  5.61  0.046
## 18 3.774 -0.03086                           +  7 -51.751 118.8  6.23  0.034
## 21 3.753                  0.01524           +  7 -52.019 119.3  6.77  0.026
## Models ranked by AICc(x)
## Random terms (all models):
##   1 | aquarium
```

```
get.models(ddAIC_S, subset=TRUE)
```

```
## $`17`
## Linear mixed model fit by REML ['lmerModLmerTest']
## Formula: Shannon ~ treatmentOrder + (1 | aquarium)
##    Data: alphaDiversitySnails
## REML criterion at convergence: 99.6048
## Random effects:
##  Groups   Name        Std.Dev.
##  aquarium (Intercept) 0.2195
##  Residual             0.3420
## Number of obs: 94, groups:  aquarium, 46
## Fixed Effects:
##          (Intercept)     treatmentOrderlow  treatmentOrdermedium
##               3.7550                0.1813                0.4614
##  treatmentOrderxHigh
##               0.3066
##
## $`25`
## Linear mixed model fit by REML ['lmerModLmerTest']
## Formula: Shannon ~ scaledThig + treatmentOrder + (1 | aquarium)
##    Data: alphaDiversitySnails
## REML criterion at convergence: 100.7712
## Random effects:
##  Groups   Name        Std.Dev.
##  aquarium (Intercept) 0.2008
##  Residual             0.3434
## Number of obs: 94, groups:  aquarium, 46
## Fixed Effects:
##          (Intercept)            scaledThig     treatmentOrderlow
##              3.75953               0.07368               0.16574
## treatmentOrdermedium   treatmentOrderxHigh
##              0.47017               0.28962
##
## $`19`
## Linear mixed model fit by REML ['lmerModLmerTest']
## Formula: Shannon ~ scaledmetRate + treatmentOrder + (1 | aquarium)
##    Data: alphaDiversitySnails
## REML criterion at convergence: 102.8816
## Random effects:
##  Groups   Name        Std.Dev.
##  aquarium (Intercept) 0.2290
##  Residual             0.3379
## Number of obs: 94, groups:  aquarium, 46
## Fixed Effects:
##          (Intercept)         scaledmetRate     treatmentOrderlow
##              3.77257               0.04742               0.16943
## treatmentOrdermedium   treatmentOrderxHigh
##              0.43704               0.27805
##
## $`18`
## Linear mixed model fit by REML ['lmerModLmerTest']
## Formula: Shannon ~ scaledBiteRate + treatmentOrder + (1 | aquarium)
##    Data: alphaDiversitySnails
## REML criterion at convergence: 103.5021
## Random effects:
##  Groups   Name        Std.Dev.
```

```
##  aquarium (Intercept) 0.2148
##  Residual             0.3452
## Number of obs: 94, groups:  aquarium, 46
## Fixed Effects:
##          (Intercept)        scaledBiteRate     treatmentOrderlow
##              3.77358              -0.03086               0.17215
## treatmentOrdermedium   treatmentOrderxHigh
##              0.43791               0.27159
##
## $`21`
## Linear mixed model fit by REML ['lmerModLmerTest']
## Formula: Shannon ~ scaledSpeed + treatmentOrder + (1 | aquarium)
##    Data: alphaDiversitySnails
## REML criterion at convergence: 104.0373
## Random effects:
##  Groups   Name        Std.Dev.
##  aquarium (Intercept) 0.2199
##  Residual             0.3439
## Number of obs: 94, groups:  aquarium, 46
## Fixed Effects:
##          (Intercept)           scaledSpeed     treatmentOrderlow
##              3.75293               0.01524               0.18290
## treatmentOrdermedium   treatmentOrderxHigh
##              0.46711               0.30538
##
## attr(,"rank")
## function (x)
## do.call("rank", list(x))
## <environment: 0x0000021a756aedb0>
## attr(,"call")
## AICc(x)
## attr(,"class")
## [1] "function"     "rankFunction"
## attr(,"beta")
## [1] "none"
```

```
ddMAc<-model.avg(ddAIC_S, subset= delta <7)
summary(ddMAc)
```

```
##
## Call:
## model.avg(object = ddAIC_S, subset = delta < 7)
##
## Component model call:
## lmer(formula = Shannon ~ <5 unique rhs>, data = alphaDiversitySnails,
##      na.action = na.fail)
##
## Component models:
##    df logLik   AICc delta weight
## 5   6 -49.80 112.57  0.00   0.76
## 45  7 -50.39 116.07  3.50   0.13
## 25  7 -51.44 118.18  5.61   0.05
## 15  7 -51.75 118.80  6.23   0.03
## 35  7 -52.02 119.34  6.77   0.03
##
## Term codes:
## scaledBiteRate  scaledmetRate    scaledSpeed     scaledThig treatmentOrder
##              1              2              3              4              5
##
## Model-averaged coefficients:
## (full average)
##                         Estimate Std. Error Adjusted SE z value Pr(>|z|)
## (Intercept)            3.7570045  0.0985995   0.0999745  37.580  < 2e-16 ***
## treatmentOrderlow      0.1784445  0.1425961   0.1445863   1.234  0.21714
## treatmentOrdermedium   0.4607763  0.1390980   0.1410369   3.267  0.00109 **
## treatmentOrderxHigh    0.3017943  0.1351256   0.1370051   2.203  0.02761 *
## scaledThig             0.0097428  0.0286678   0.0287661   0.339  0.73484
## scaledmetRate          0.0021830  0.0136882   0.0137798   0.158  0.87413
## scaledBiteRate        -0.0010418  0.0100162   0.0101139   0.103  0.91796
## scaledSpeed            0.0003936  0.0069620   0.0070484   0.056  0.95547
##
## (conditional average)
##                       Estimate Std. Error Adjusted SE z value Pr(>|z|)
## (Intercept)            3.75700    0.09860     0.09997  37.580  < 2e-16 ***
## treatmentOrderlow      0.17844    0.14260     0.14459   1.234  0.21714
## treatmentOrdermedium   0.46078    0.13910     0.14104   3.267  0.00109 **
## treatmentOrderxHigh    0.30179    0.13513     0.13701   2.203  0.02761 *
## scaledThig             0.07368    0.03878     0.03933   1.873  0.06102 .
## scaledmetRate          0.04742    0.04387     0.04449   1.066  0.28648
## scaledBiteRate        -0.03086    0.04530     0.04594   0.672  0.50164
## scaledSpeed            0.01524    0.04062     0.04120   0.370  0.71147
## ---
## Signif. codes:  0 '***' 0.001 '**' 0.01 '*' 0.05 '.' 0.1 ' ' 1
```

```
rm(dd, ddAIC_S, ddMAc)

# chao1

model2<-lmer(Chao1~scaledSpeed*treatmentOrder + scaledThig*treatmentOrder + scaledmetRate*treat
mentOrder+ scaledBiteRate*treatmentOrder +treatmentOrder+ (1|aquarium), data=alphaDiversitySnai
ls, na.action=na.fail)
summary(model2)
```

```
## Linear mixed model fit by REML. t-tests use Satterthwaite's method [
## lmerModLmerTest]
## Formula: Chao1 ~ scaledSpeed * treatmentOrder + scaledThig * treatmentOrder +
##     scaledmetRate * treatmentOrder + scaledBiteRate * treatmentOrder +
##     treatmentOrder + (1 | aquarium)
##    Data: alphaDiversitySnails
##
## REML criterion at convergence: 1118.6
##
## Scaled residuals:
##     Min      1Q  Median      3Q     Max
## -1.7213 -0.6180 -0.1688  0.3949  3.4344
##
## Random effects:
##  Groups   Name        Variance Std.Dev.
##  aquarium (Intercept)   797.5   28.24
##  Residual             96886.7  311.27
## Number of obs: 94, groups:  aquarium, 46
##
## Fixed effects:
##                                  Estimate Std. Error       df t value
## (Intercept)                       597.295     83.767   52.003   7.130
## scaledSpeed                        -6.408     71.491   73.664  -0.090
## treatmentOrderlow                 -21.965    114.732   49.487  -0.191
## treatmentOrdermedium              170.050    109.707   44.012   1.550
## treatmentOrderxHigh                46.127    110.978   42.990   0.416
## scaledThig                         80.115     89.986   73.663   0.890
## scaledmetRate                      -9.905     87.632   73.983  -0.113
## scaledBiteRate                     -5.433     71.672   73.767  -0.076
## scaledSpeed:treatmentOrderlow     -31.325    125.804   67.662  -0.249
## scaledSpeed:treatmentOrdermedium  161.488    102.149   72.656   1.581
## scaledSpeed:treatmentOrderxHigh   -18.609    100.959   72.280  -0.184
## treatmentOrderlow:scaledThig       26.832    117.500   72.770   0.228
## treatmentOrdermedium:scaledThig    19.322    120.979   73.959   0.160
## treatmentOrderxHigh:scaledThig    -24.745    117.652   73.925  -0.210
## treatmentOrderlow:scaledmetRate   -46.855    110.141   72.956  -0.425
## treatmentOrdermedium:scaledmetRate  94.729  116.973   74.000   0.810
## treatmentOrderxHigh:scaledmetRate -123.250  124.655   69.739  -0.989
## treatmentOrderlow:scaledBiteRate   19.375    122.499   70.207   0.158
## treatmentOrdermedium:scaledBiteRate -91.362  116.991   73.884  -0.781
## treatmentOrderxHigh:scaledBiteRate  53.037   102.633   71.293   0.517
##                                  Pr(>|t|)
## (Intercept)                      3.06e-09 ***
## scaledSpeed                         0.929
## treatmentOrderlow                   0.849
## treatmentOrdermedium                0.128
## treatmentOrderxHigh                 0.680
## scaledThig                          0.376
## scaledmetRate                       0.910
## scaledBiteRate                      0.940
## scaledSpeed:treatmentOrderlow       0.804
## scaledSpeed:treatmentOrdermedium    0.118
## scaledSpeed:treatmentOrderxHigh     0.854
## treatmentOrderlow:scaledThig        0.820
## treatmentOrdermedium:scaledThig     0.874
```

```
## treatmentOrderxHigh:scaledThig         0.834
## treatmentOrderlow:scaledmetRate        0.672
## treatmentOrdermedium:scaledmetRate     0.421
## treatmentOrderxHigh:scaledmetRate      0.326
## treatmentOrderlow:scaledBiteRate       0.875
## treatmentOrdermedium:scaledBiteRate    0.437
## treatmentOrderxHigh:scaledBiteRate     0.607
## ---
## Signif. codes:  0 '***' 0.001 '**' 0.01 '*' 0.05 '.' 0.1 ' ' 1
```

```
##
## Correlation matrix not shown by default, as p = 20 > 12.
## Use print(x, correlation=TRUE)  or
##     vcov(x)        if you need it
```

```
model2<-lmer(Chao1~scaledSpeed + scaledThig + scaledmetRate+ scaledBiteRate +treatmentOrder+ (1|aquarium), data=alphaDiversitySnails, na.action=na.fail)
```

```
## boundary (singular) fit: see help('isSingular')
```

```
summary(model2)
```

```
## Linear mixed model fit by REML. t-tests use Satterthwaite's method [
## lmerModLmerTest]
## Formula: Chao1 ~ scaledSpeed + scaledThig + scaledmetRate + scaledBiteRate +
##     treatmentOrder + (1 | aquarium)
##    Data: alphaDiversitySnails
##
## REML criterion at convergence: 1260.4
##
## Scaled residuals:
##     Min      1Q  Median      3Q     Max
## -1.4630 -0.6660 -0.1904  0.4812  3.5827
##
## Random effects:
##  Groups   Name        Variance Std.Dev.
##  aquarium (Intercept)     0      0.0
##  Residual             95536    309.1
## Number of obs: 94, groups:  aquarium, 46
##
## Fixed effects:
##                      Estimate Std. Error       df t value Pr(>|t|)
## (Intercept)          577.8050    73.5052  86.0000   7.861 1.01e-11 ***
## scaledSpeed           20.4986    34.9952  86.0000   0.586   0.5596
## scaledThig            50.8947    35.2133  86.0000   1.445   0.1520
## scaledmetRate        -35.7522    35.2867  86.0000  -1.013   0.3138
## scaledBiteRate         0.3146    37.6821  86.0000   0.008   0.9934
## treatmentOrderlow      9.9219    96.9460  86.0000   0.102   0.9187
## treatmentOrdermedium 187.7763   101.5574  86.0000   1.849   0.0679 .
## treatmentOrderxHigh   13.5906   101.7547  86.0000   0.134   0.8941
## ---
## Signif. codes:  0 '***' 0.001 '**' 0.01 '*' 0.05 '.' 0.1 ' ' 1
##
## Correlation of Fixed Effects:
##             (Intr) scldSp scldTh scldmR scldBR trtmntOrdrl trtmntOrdrm
## scaledSpeed -0.150
## scaledThig   0.012 -0.196
## scaledmetRt  0.189 -0.154 -0.295
## scaledBitRt -0.364  0.212  0.109 -0.180
## trtmntOrdrl -0.703  0.082 -0.079 -0.065  0.133
## trtmntOrdrm -0.772  0.198  0.035 -0.203  0.378  0.525
## trtmntOrdrH -0.797  0.114 -0.012 -0.221  0.446  0.537       0.631
## optimizer (nloptwrap) convergence code: 0 (OK)
## boundary (singular) fit: see help('isSingular')
```

```
dd<-dredge(model2, subset= ~treatmentOrder, evaluate=TRUE, rank=AICc)
```

```
## Warning in dredge(model2, subset = ~treatmentOrder, evaluate = TRUE, rank =
## AICc): comparing models fitted by REML
```

```
## Fixed term is "(Intercept)"
## boundary (singular) fit: see help('isSingular')
## boundary (singular) fit: see help('isSingular')
## boundary (singular) fit: see help('isSingular')
## boundary (singular) fit: see help('isSingular')
## boundary (singular) fit: see help('isSingular')
## boundary (singular) fit: see help('isSingular')
## boundary (singular) fit: see help('isSingular')
## boundary (singular) fit: see help('isSingular')
```

```
dd
```

```
## Global model call: lmer(formula = Chao1 ~ scaledSpeed + scaledThig + scaledmetRate +
##     scaledBiteRate + treatmentOrder + (1 | aquarium), data = alphaDiversitySnails,
##     na.action = na.fail)
## ---
## Model selection table
##    (Intrc)    sclBR  scldR scldS scldT trtmO df   logLik   AICc delta weight
## 32   577.8   0.3146 -35.75 20.50 50.89     + 10 -630.178 1283.0  0.00  0.898
## 31   578.0          -35.70 20.44 50.86     +  9 -634.724 1289.6  6.58  0.033
## 28   584.3  -4.3690 -32.57       54.94     +  9 -634.823 1289.8  6.78  0.030
## 30   591.9  -6.5410        15.04 40.37     +  9 -635.174 1290.5  7.48  0.021
## 24   577.1  -5.2600 -20.75 30.52           +  9 -635.696 1291.5  8.53  0.013
## 27   581.3          -33.20       55.58     +  8 -639.348 1296.4 13.38  0.001
## 29   587.5                 16.20 40.73     +  8 -639.719 1297.1 14.12  0.001
## 26   595.8  -9.6030              44.12     +  8 -639.729 1297.2 14.14  0.001
## 23   573.4          -21.48 31.68           +  8 -640.251 1298.2 15.19  0.000
## 22   586.0  -8.9420        25.76           +  8 -640.327 1298.3 15.34  0.000
## 20   586.8 -13.3700 -14.00                 +  8 -640.547 1298.8 15.78  0.000
## 25   589.6                       45.09     +  7 -644.271 1303.8 20.84  0.000
## 21   580.2                 27.51           +  7 -644.885 1305.1 22.07  0.000
## 18   591.8 -15.1700                        +  7 -645.051 1305.4 22.40  0.000
## 19   577.9          -15.31                 +  7 -645.128 1305.6 22.55  0.000
## 17   582.2                                 +  6 -649.644 1312.3 29.25  0.000
## Models ranked by AICc(x)
## Random terms (all models):
##   1 | aquarium
```

```
ddAIC_C<-subset(dd, delta < 7)
ddAIC_C
```

```
## Global model call: lmer(formula = Chao1 ~ scaledSpeed + scaledThig + scaledmetRate +
##     scaledBiteRate + treatmentOrder + (1 | aquarium), data = alphaDiversitySnails,
##     na.action = na.fail)
## ---
## Model selection table
##    (Intrc)   sclBR  scldR scldS scldT trtmO df   logLik   AICc delta weight
## 32   577.8  0.3146 -35.75 20.50 50.89     + 10 -630.178 1283.0  0.00  0.934
## 31   578.0         -35.70 20.44 50.86     +  9 -634.724 1289.6  6.58  0.035
## 28   584.3 -4.3690 -32.57       54.94     +  9 -634.823 1289.8  6.78  0.031
## Models ranked by AICc(x)
## Random terms (all models):
##   1 | aquarium
```

```
get.models(ddAIC_C, subset=TRUE)
```

```
## boundary (singular) fit: see help('isSingular')
## boundary (singular) fit: see help('isSingular')
## boundary (singular) fit: see help('isSingular')
```

```
## $`32`
## Linear mixed model fit by REML ['lmerModLmerTest']
## Formula: Chao1 ~ scaledBiteRate + scaledmetRate + scaledSpeed + scaledThig +
##     treatmentOrder + (1 | aquarium)
##    Data: alphaDiversitySnails
## REML criterion at convergence: 1260.357
## Random effects:
##  Groups   Name        Std.Dev.
##  aquarium (Intercept)   0.0
##  Residual             309.1
## Number of obs: 94, groups:  aquarium, 46
## Fixed Effects:
##         (Intercept)        scaledBiteRate         scaledmetRate
##            577.8050                0.3146              -35.7522
##         scaledSpeed            scaledThig     treatmentOrderlow
##             20.4986               50.8947                9.9219
## treatmentOrdermedium   treatmentOrderxHigh
##            187.7763               13.5906
## optimizer (nloptwrap) convergence code: 0 (OK) ; 0 optimizer warnings; 1 lme4 warnings
##
## $`31`
## Linear mixed model fit by REML ['lmerModLmerTest']
## Formula: Chao1 ~ scaledmetRate + scaledSpeed + scaledThig + treatmentOrder +
##     (1 | aquarium)
##    Data: alphaDiversitySnails
## REML criterion at convergence: 1269.447
## Random effects:
##  Groups   Name        Std.Dev.
##  aquarium (Intercept)   0.0
##  Residual             307.3
## Number of obs: 94, groups:  aquarium, 46
## Fixed Effects:
##         (Intercept)         scaledmetRate           scaledSpeed
##             578.028               -35.699                20.437
##          scaledThig     treatmentOrderlow  treatmentOrdermedium
##              50.863                 9.814               187.456
##  treatmentOrderxHigh
##              13.212
## optimizer (nloptwrap) convergence code: 0 (OK) ; 0 optimizer warnings; 1 lme4 warnings
##
## $`28`
## Linear mixed model fit by REML ['lmerModLmerTest']
## Formula:
## Chao1 ~ scaledBiteRate + scaledmetRate + scaledThig + treatmentOrder +
##     (1 | aquarium)
##    Data: alphaDiversitySnails
## REML criterion at convergence: 1269.645
## Random effects:
##  Groups   Name        Std.Dev.
##  aquarium (Intercept)   0.0
##  Residual             307.9
## Number of obs: 94, groups:  aquarium, 46
## Fixed Effects:
##         (Intercept)        scaledBiteRate         scaledmetRate
##             584.263                -4.369               -32.570
```

```
##           scaledThig     treatmentOrderlow  treatmentOrdermedium
##               54.939                 5.253               175.981
##  treatmentOrderxHigh
##                6.810
## optimizer (nloptwrap) convergence code: 0 (OK) ; 0 optimizer warnings; 1 lme4 warnings
##
## attr(,"rank")
## function (x)
## do.call("rank", list(x))
## <environment: 0x0000021a0565e8f0>
## attr(,"call")
## AICc(x)
## attr(,"class")
## [1] "function"     "rankFunction"
## attr(,"beta")
## [1] "none"
```

```
ddMAc<-model.avg(ddAIC_C, subset= delta <7)
summary(ddMAc)
```

```
##
## Call:
## model.avg(object = ddAIC_C, subset = delta < 7)
##
## Component model call:
## lmer(formula = Chao1 ~ <3 unique rhs>, data = alphaDiversitySnails,
##      na.action = na.fail)
##
## Component models:
##       df  logLik    AICc delta weight
## 12345 10 -630.18 1283.01  0.00   0.93
## 2345   9 -634.72 1289.59  6.58   0.03
## 1245   9 -634.82 1289.79  6.78   0.03
##
## Term codes:
## scaledBiteRate  scaledmetRate    scaledSpeed     scaledThig treatmentOrder
##              1              2              3              4              5
##
## Model-averaged coefficients:
## (full average)
##                      Estimate Std. Error Adjusted SE z value Pr(>|z|)
## (Intercept)          578.0159    73.2970     74.3672   7.772   <2e-16 ***
## scaledBiteRate         0.1563    36.9993     37.5396   0.004   0.9967
## scaledmetRate        -35.6503    35.2473     35.7619   0.997   0.3188
## scaledSpeed           19.8516    34.5913     35.0913   0.566   0.5716
## scaledThig            51.0208    35.1808     35.6944   1.429   0.1529
## treatmentOrderlow      9.7713    96.8789     98.2936   0.099   0.9208
## treatmentOrdermedium 187.3941   101.2342    102.7121   1.824   0.0681 .
## treatmentOrderxHigh   13.3642   101.3606    102.8407   0.130   0.8966
##
## (conditional average)
##                      Estimate Std. Error Adjusted SE z value Pr(>|z|)
## (Intercept)           578.016     73.297      74.367   7.772   <2e-16 ***
## scaledBiteRate          0.162     37.659      38.209   0.004   0.9966
## scaledmetRate         -35.650     35.247      35.762   0.997   0.3188
## scaledSpeed            20.496     34.960      35.471   0.578   0.5634
## scaledThig             51.021     35.181      35.694   1.429   0.1529
## treatmentOrderlow       9.771     96.879      98.294   0.099   0.9208
## treatmentOrdermedium  187.394    101.234     102.712   1.824   0.0681 .
## treatmentOrderxHigh    13.364    101.361     102.841   0.130   0.8966
## ---
## Signif. codes:  0 '***' 0.001 '**' 0.01 '*' 0.05 '.' 0.1 ' ' 1
```

```
# observed

model3<-lmer(Observed~scaledSpeed*treatmentOrder + scaledThig*treatmentOrder + scaledmetRate*treatmentOrder+ scaledBiteRate*treatmentOrder +treatmentOrder+ (1|aquarium), data=alphaDiversitySnails, na.action=na.fail)
summary(model3)
```

```
## Linear mixed model fit by REML. t-tests use Satterthwaite's method [
## lmerModLmerTest]
## Formula:
## Observed ~ scaledSpeed * treatmentOrder + scaledThig * treatmentOrder +
##     scaledmetRate * treatmentOrder + scaledBiteRate * treatmentOrder +
##     treatmentOrder + (1 | aquarium)
##    Data: alphaDiversitySnails
##
## REML criterion at convergence: 984.2
##
## Scaled residuals:
##     Min      1Q  Median      3Q     Max
## -2.0418 -0.6155 -0.1443  0.5071  2.6671
##
## Random effects:
##  Groups   Name        Variance Std.Dev.
##  aquarium (Intercept)  1367     36.97
##  Residual             14653    121.05
## Number of obs: 94, groups:  aquarium, 46
##
## Fixed effects:
##                                  Estimate Std. Error      df t value
## (Intercept)                       345.438     34.709  49.920   9.952
## scaledSpeed                        -4.676     28.726  73.360  -0.163
## treatmentOrderlow                  23.603     47.666  46.433   0.495
## treatmentOrdermedium               99.481     45.792  41.817   2.172
## treatmentOrderxHigh                48.975     46.369  40.829   1.056
## scaledThig                         30.454     36.399  73.274   0.837
## scaledmetRate                      -2.386     35.319  73.998  -0.068
## scaledBiteRate                      1.873     28.975  73.492   0.065
## scaledSpeed:treatmentOrderlow      -3.665     50.038  65.848  -0.073
## scaledSpeed:treatmentOrdermedium   78.948     40.880  70.515   1.931
## scaledSpeed:treatmentOrderxHigh    -3.100     40.905  73.412  -0.076
## treatmentOrderlow:scaledThig       20.727     47.110  72.660   0.440
## treatmentOrdermedium:scaledThig    26.742     48.801  73.996   0.548
## treatmentOrderxHigh:scaledThig    -18.059     47.462  73.999  -0.380
## treatmentOrderlow:scaledmetRate   -29.486     44.636  72.600  -0.661
## treatmentOrdermedium:scaledmetRate  44.725     47.124  73.911   0.949
## treatmentOrderxHigh:scaledmetRate  -53.316     50.770  69.549  -1.050
## treatmentOrderlow:scaledBiteRate   10.650     48.893  69.493   0.218
## treatmentOrdermedium:scaledBiteRate -48.916     47.178  73.957  -1.037
## treatmentOrderxHigh:scaledBiteRate   7.046     41.669  72.338   0.169
##                                  Pr(>|t|)
## (Intercept)                      1.92e-13 ***
## scaledSpeed                        0.8711
## treatmentOrderlow                  0.6228
## treatmentOrdermedium               0.0355 *
## treatmentOrderxHigh                0.2971
## scaledThig                         0.4055
## scaledmetRate                      0.9463
## scaledBiteRate                     0.9486
## scaledSpeed:treatmentOrderlow      0.9418
## scaledSpeed:treatmentOrdermedium   0.0575 .
## scaledSpeed:treatmentOrderxHigh    0.9398
## treatmentOrderlow:scaledThig       0.6613
```

```
## treatmentOrdermedium:scaledThig       0.5853
## treatmentOrderxHigh:scaledThig        0.7047
## treatmentOrderlow:scaledmetRate       0.5110
## treatmentOrdermedium:scaledmetRate    0.3457
## treatmentOrderxHigh:scaledmetRate     0.2973
## treatmentOrderlow:scaledBiteRate      0.8282
## treatmentOrdermedium:scaledBiteRate   0.3032
## treatmentOrderxHigh:scaledBiteRate    0.8662
## ---
## Signif. codes:  0 '***' 0.001 '**' 0.01 '*' 0.05 '.' 0.1 ' ' 1
```

```
##
## Correlation matrix not shown by default, as p = 20 > 12.
## Use print(x, correlation=TRUE)  or
##     vcov(x)        if you need it
```

```
model3<-lmer(Observed~scaledSpeed + scaledThig + scaledmetRate+ scaledBiteRate +treatmentOrder+
(1|aquarium), data=alphaDiversitySnails, na.action=na.fail)
summary(model3)
```

```
## Linear mixed model fit by REML. t-tests use Satterthwaite's method [
## lmerModLmerTest]
## Formula:
## Observed ~ scaledSpeed + scaledThig + scaledmetRate + scaledBiteRate +
##     treatmentOrder + (1 | aquarium)
##    Data: alphaDiversitySnails
##
## REML criterion at convergence: 1107.6
##
## Scaled residuals:
##     Min      1Q  Median      3Q     Max
## -1.7907 -0.7048 -0.1172  0.5325  2.6935
##
## Random effects:
##  Groups   Name        Variance Std.Dev.
##  aquarium (Intercept)   598.1   24.46
##  Residual             15627.7  125.01
## Number of obs: 94, groups:  aquarium, 46
##
## Fixed effects:
##                       Estimate Std. Error       df t value Pr(>|t|)
## (Intercept)           338.1812    30.7308  51.5963  11.005 3.82e-15 ***
## scaledSpeed            10.1495    14.3928  85.7930   0.705   0.4826
## scaledThig             21.9088    14.4597  84.6334   1.515   0.1335
## scaledmetRate         -16.6005    14.6652  69.0770  -1.132   0.2616
## scaledBiteRate         -0.1211    15.5019  85.9330  -0.008   0.9938
## treatmentOrderlow      39.2372    40.7496  39.1903   0.963   0.3415
## treatmentOrdermedium 105.4771    42.5884  44.2274   2.477   0.0172 *
## treatmentOrderxHigh    38.7349    42.6525  45.1717   0.908   0.3686
## ---
## Signif. codes:  0 '***' 0.001 '**' 0.01 '*' 0.05 '.' 0.1 ' ' 1
##
## Correlation of Fixed Effects:
##             (Intr) scldSp scldTh scldmR scldBR trtmntOrdrl trtmntOrdrm
## scaledSpeed -0.150
## scaledThig   0.007 -0.201
## scaledmetRt  0.188 -0.144 -0.300
## scaledBitRt -0.359  0.214  0.106 -0.178
## trtmntOrdrl -0.701  0.082 -0.075 -0.064  0.132
## trtmntOrdrm -0.768  0.196  0.039 -0.202  0.369  0.522
## trtmntOrdrH -0.793  0.111 -0.007 -0.219  0.439  0.534       0.623
```

```
dd<-dredge(model3, subset= ~treatmentOrder, evaluate=TRUE, rank=AICc)
```

```
## Warning in dredge(model3, subset = ~treatmentOrder, evaluate = TRUE, rank =
## AICc): comparing models fitted by REML
```

```
## Fixed term is "(Intercept)"
```

```
dd
```

```
## Global model call: lmer(formula = Observed ~ scaledSpeed + scaledThig + scaledmetRate +
##     scaledBiteRate + treatmentOrder + (1 | aquarium), data = alphaDiversitySnails,
##     na.action = na.fail)
## ---
## Model selection table
##    (Intrc)   sclBR   scldR  scldS scldT trtmO df   logLik   AICc delta weight
## 32   338.2 -0.1211 -16.600 10.150 21.91     + 10 -553.806 1130.3  0.00  0.787
## 31   338.1         -16.620 10.170 21.91     +  9 -557.463 1135.1  4.81  0.071
## 28   341.4 -2.4850 -15.110        23.99     +  9 -557.639 1135.4  5.16  0.060
## 30   344.8 -3.1890          7.832 16.94     +  9 -558.050 1136.2  5.98  0.040
## 24   338.7 -2.1150  -9.842 14.610           +  9 -558.517 1137.2  6.91  0.025
## 27   339.8         -15.470        24.31     +  8 -561.283 1140.3 10.00  0.005
## 29   342.8                  8.436 17.02     +  8 -561.714 1141.1 10.86  0.003
## 26   346.7 -4.8970                18.99     +  8 -561.775 1141.2 10.98  0.003
## 23   337.3         -10.110 15.070           +  8 -562.184 1142.1 11.80  0.002
## 22   343.0 -3.7230         12.530           +  8 -562.328 1142.3 12.09  0.002
## 20   343.4 -6.0480  -6.746                  +  8 -562.617 1142.9 12.67  0.001
## 25   343.8                        19.39     +  7 -565.448 1146.2 15.94  0.000
## 21   340.8                 13.280           +  7 -566.000 1147.3 17.04  0.000
## 18   346.0 -6.8750                          +  7 -566.282 1147.9 17.60  0.000
## 19   339.6          -7.306                  +  7 -566.325 1148.0 17.69  0.000
## 17   341.9                                  +  6 -570.004 1153.0 22.71  0.000
## Models ranked by AICc(x)
## Random terms (all models):
##   1 | aquarium
```

```
ddAIC_C<-subset(dd, delta < 7)
ddAIC_C
```

```
## Global model call: lmer(formula = Observed ~ scaledSpeed + scaledThig + scaledmetRate +
##     scaledBiteRate + treatmentOrder + (1 | aquarium), data = alphaDiversitySnails,
##     na.action = na.fail)
## ---
## Model selection table
##    (Intrc)   sclBR   scldR  scldS scldT trtmO df   logLik   AICc delta weight
## 32   338.2 -0.1211 -16.600 10.150 21.91     + 10 -553.806 1130.3  0.00  0.801
## 31   338.1         -16.620 10.170 21.91     +  9 -557.463 1135.1  4.81  0.072
## 28   341.4 -2.4850 -15.110        23.99     +  9 -557.639 1135.4  5.16  0.061
## 30   344.8 -3.1890          7.832 16.94     +  9 -558.050 1136.2  5.98  0.040
## 24   338.7 -2.1150  -9.842 14.610           +  9 -558.517 1137.2  6.91  0.025
## Models ranked by AICc(x)
## Random terms (all models):
##   1 | aquarium
```

```
get.models(ddAIC_C, subset=TRUE)
```

```
## $`32`
## Linear mixed model fit by REML ['lmerModLmerTest']
## Formula: 
## Observed ~ scaledBiteRate + scaledmetRate + scaledSpeed + scaledThig +  
##     treatmentOrder + (1 | aquarium)
##    Data: alphaDiversitySnails
## REML criterion at convergence: 1107.611
## Random effects:
##  Groups   Name        Std.Dev.
##  aquarium (Intercept)  24.46  
##  Residual             125.01  
## Number of obs: 94, groups:  aquarium, 46
## Fixed Effects:
##          (Intercept)        scaledBiteRate         scaledmetRate  
##             338.1812               -0.1211              -16.6005  
##          scaledSpeed            scaledThig     treatmentOrderlow  
##              10.1495               21.9088               39.2372  
## treatmentOrdermedium   treatmentOrderxHigh  
##             105.4771               38.7349  
## 
## $`31`
## Linear mixed model fit by REML ['lmerModLmerTest']
## Formula: 
## Observed ~ scaledmetRate + scaledSpeed + scaledThig + treatmentOrder +  
##     (1 | aquarium)
##    Data: alphaDiversitySnails
## REML criterion at convergence: 1114.926
## Random effects:
##  Groups   Name        Std.Dev.
##  aquarium (Intercept)  24.46  
##  Residual             124.27  
## Number of obs: 94, groups:  aquarium, 46
## Fixed Effects:
##          (Intercept)         scaledmetRate           scaledSpeed  
##               338.11                -16.62                 10.17  
##           scaledThig     treatmentOrderlow  treatmentOrdermedium  
##                21.91                 39.28                105.57  
##  treatmentOrderxHigh  
##                38.86  
## 
## $`28`
## Linear mixed model fit by REML ['lmerModLmerTest']
## Formula: 
## Observed ~ scaledBiteRate + scaledmetRate + scaledThig + treatmentOrder +  
##     (1 | aquarium)
##    Data: alphaDiversitySnails
## REML criterion at convergence: 1115.278
## Random effects:
##  Groups   Name        Std.Dev.
##  aquarium (Intercept)  23.48  
##  Residual             124.80  
## Number of obs: 94, groups:  aquarium, 46
## Fixed Effects:
##          (Intercept)        scaledBiteRate         scaledmetRate  
##              341.361                -2.485               -15.107  
```

```
##          scaledThig     treatmentOrderlow  treatmentOrdermedium
##              23.988                36.892                99.774
##  treatmentOrderxHigh
##              35.469
##
## $`30`
## Linear mixed model fit by REML ['lmerModLmerTest']
## Formula:
## Observed ~ scaledBiteRate + scaledSpeed + scaledThig + treatmentOrder +
##     (1 | aquarium)
##    Data: alphaDiversitySnails
## REML criterion at convergence: 1116.1
## Random effects:
##  Groups   Name        Std.Dev.
##  aquarium (Intercept)  25.9
##  Residual             125.0
## Number of obs: 94, groups:  aquarium, 46
## Fixed Effects:
##         (Intercept)        scaledBiteRate           scaledSpeed
##             344.818                -3.189                 7.832
##          scaledThig     treatmentOrderlow  treatmentOrdermedium
##              16.941                36.248                95.496
##  treatmentOrderxHigh
##              28.039
##
## $`24`
## Linear mixed model fit by REML ['lmerModLmerTest']
## Formula:
## Observed ~ scaledBiteRate + scaledmetRate + scaledSpeed + treatmentOrder +
##     (1 | aquarium)
##    Data: alphaDiversitySnails
## REML criterion at convergence: 1117.033
## Random effects:
##  Groups   Name        Std.Dev.
##  aquarium (Intercept)  33.85
##  Residual             124.03
## Number of obs: 94, groups:  aquarium, 46
## Fixed Effects:
##         (Intercept)        scaledBiteRate         scaledmetRate
##             338.658                -2.115                -9.842
##         scaledSpeed     treatmentOrderlow  treatmentOrdermedium
##              14.606                43.549               100.875
##  treatmentOrderxHigh
##              38.137
##
## attr(,"rank")
## function (x)
## do.call("rank", list(x))
## <environment: 0x0000021a080c5a60>
## attr(,"call")
## AICc(x)
## attr(,"class")
## [1] "function"     "rankFunction"
## attr(,"beta")
## [1] "none"
```

```
ddMAc<-model.avg(ddAIC_C, subset= delta <7)
summary(ddMAc)
```

```
##
## Call:
## model.avg(object = ddAIC_C, subset = delta < 7)
##
## Component model call:
## lmer(formula = Observed ~ <5 unique rhs>, data = alphaDiversitySnails,
##      na.action = na.fail)
##
## Component models:
##       df  logLik    AICc delta weight
## 12345 10 -553.81 1130.26  0.00   0.80
## 2345   9 -557.46 1135.07  4.81   0.07
## 1245   9 -557.64 1135.42  5.16   0.06
## 1345   9 -558.05 1136.24  5.98   0.04
## 1235   9 -558.52 1137.18  6.91   0.03
##
## Term codes:
## scaledBiteRate  scaledmetRate    scaledSpeed     scaledThig treatmentOrder
##              1              2              3              4              5
##
## Model-averaged coefficients:
## (full average)
##                      Estimate Std. Error Adjusted SE z value Pr(>|z|)
## (Intercept)          338.6484    30.5820     31.0270  10.915   <2e-16 ***
## scaledBiteRate        -0.4298    14.9201     15.1371   0.028   0.9773
## scaledmetRate        -15.6719    14.7157     14.9190   1.050   0.2935
## scaledSpeed            9.5540    14.1434     14.3430   0.666   0.5053
## scaledThig            21.2820    14.6673     14.8684   1.431   0.1523
## treatmentOrderlow     39.0861    40.7301     41.3236   0.946   0.3442
## treatmentOrdermedium 104.6191    42.3674     42.9833   2.434   0.0149 *
## treatmentOrderxHigh   38.1000    42.3605     42.9766   0.887   0.3753
##
## (conditional average)
##                      Estimate Std. Error Adjusted SE z value Pr(>|z|)
## (Intercept)          338.6484    30.5820     31.0270  10.915   <2e-16 ***
## scaledBiteRate        -0.4634    15.4914     15.7168   0.029   0.9765
## scaledmetRate        -16.3295    14.6595     14.8721   1.098   0.2722
## scaledSpeed           10.1719    14.3766     14.5856   0.697   0.4856
## scaledThig            21.8335    14.4452     14.6546   1.490   0.1363
## treatmentOrderlow     39.0861    40.7301     41.3236   0.946   0.3442
## treatmentOrdermedium 104.6191    42.3674     42.9833   2.434   0.0149 *
## treatmentOrderxHigh   38.1000    42.3605     42.9766   0.887   0.3753
## ---
## Signif. codes:  0 '***' 0.001 '**' 0.01 '*' 0.05 '.' 0.1 ' ' 1
```

```
rm(dd, ddAIC_C, ddMAc, model1, model2, model3)
```

can carrot juice consumptions explain alpha

diversity or treatment on palatability?

```
###Can alpha diversity increase be explained by consumption of carrot juice?

model1<-lmer(Shannon~Pretraining+ (1|aquarium), data=alphaDiversitySnails)
summary(model1)
```

```
## Linear mixed model fit by REML. t-tests use Satterthwaite's method [
## lmerModLmerTest]
## Formula: Shannon ~ Pretraining + (1 | aquarium)
##    Data: alphaDiversitySnails
##
## REML criterion at convergence: 111.1
##
## Scaled residuals:
##     Min      1Q  Median      3Q     Max
## -3.5191 -0.3760  0.1103  0.5080  1.9157
##
## Random effects:
##  Groups   Name        Variance Std.Dev.
##  aquarium (Intercept) 0.07545  0.2747
##  Residual             0.11446  0.3383
## Number of obs: 94, groups:  aquarium, 46
##
## Fixed effects:
##              Estimate Std. Error        df t value Pr(>|t|)
## (Intercept)  4.020000   0.117524 90.537245  34.206   <2e-16 ***
## Pretraining -0.001601   0.006802 78.056065  -0.235    0.815
## ---
## Signif. codes:  0 '***' 0.001 '**' 0.01 '*' 0.05 '.' 0.1 ' ' 1
##
## Correlation of Fixed Effects:
##             (Intr)
## Pretraining -0.885
```

```
model2<-lmer(Shannon~Posttraining+ (1|aquarium), data=alphaDiversitySnails)
summary(model2)
```

```
## Linear mixed model fit by REML. t-tests use Satterthwaite's method [
## lmerModLmerTest]
## Formula: Shannon ~ Posttraining + (1 | aquarium)
##    Data: alphaDiversitySnails
##
## REML criterion at convergence: 110
##
## Scaled residuals:
##     Min      1Q  Median      3Q     Max
## -3.5283 -0.3988  0.1410  0.5517  2.1217
##
## Random effects:
##  Groups   Name        Variance Std.Dev.
##  aquarium (Intercept) 0.07167  0.2677
##  Residual             0.11392  0.3375
## Number of obs: 94, groups:  aquarium, 46
##
## Fixed effects:
##               Estimate Std. Error        df t value Pr(>|t|)
## (Intercept)   3.915596   0.082080 85.340487  47.705   <2e-16 ***
## Posttraining  0.007075   0.005482 88.779165   1.291      0.2
## ---
## Signif. codes:  0 '***' 0.001 '**' 0.01 '*' 0.05 '.' 0.1 ' ' 1
##
## Correlation of Fixed Effects:
##             (Intr)
## Posttrainng -0.755
```

```
alphaDiversitySnails$Pretraining<-as.numeric(alphaDiversitySnails$Pretraining)
alphaDiversitySnails$Posttraining<-as.numeric(alphaDiversitySnails$Posttraining)
Alltraining2<-rowSums(alphaDiversitySnails[ , c("Pretraining", "Posttraining")])

alphaDiversitySnails<-cbind(alphaDiversitySnails, Alltraining2)

model3<-lmer(Shannon~Alltraining2+ (1|aquarium), data=alphaDiversitySnails) ###t = 0.457    p = 0.649
summary(model3)
```

```
## Linear mixed model fit by REML. t-tests use Satterthwaite's method [
## lmerModLmerTest]
## Formula: Shannon ~ Alltraining2 + (1 | aquarium)
##    Data: alphaDiversitySnails
##
## REML criterion at convergence: 111.9
##
## Scaled residuals:
##     Min      1Q  Median      3Q     Max
## -3.6061 -0.3803  0.0951  0.5237  2.0603
##
## Random effects:
##  Groups   Name        Variance Std.Dev.
##  aquarium (Intercept) 0.07689  0.2773
##  Residual             0.11286  0.3360
## Number of obs: 94, groups:  aquarium, 46
##
## Fixed effects:
##               Estimate Std. Error        df t value Pr(>|t|)
## (Intercept)   3.923283   0.112933 91.406926   34.74   <2e-16 ***
## Alltraining2  0.002712   0.003713 80.793336    0.73    0.467
## ---
## Signif. codes:  0 '***' 0.001 '**' 0.01 '*' 0.05 '.' 0.1 ' ' 1
##
## Correlation of Fixed Effects:
##             (Intr)
## Alltrainng2 -0.875
```

```
# observed

model4<-lmer(Observed~Pretraining+ (1|aquarium), data=alphaDiversitySnails)
summary(model4)
```

```
## Linear mixed model fit by REML. t-tests use Satterthwaite's method [
## lmerModLmerTest]
## Formula: Observed ~ Pretraining + (1 | aquarium)
##    Data: alphaDiversitySnails
##
## REML criterion at convergence: 1168
##
## Scaled residuals:
##     Min      1Q  Median      3Q     Max
## -1.7228 -0.7244 -0.1744  0.5999  2.5881
##
## Random effects:
##  Groups   Name        Variance Std.Dev.
##  aquarium (Intercept)  2629     51.27
##  Residual             14311    119.63
## Number of obs: 94, groups:  aquarium, 46
##
## Fixed effects:
##             Estimate Std. Error      df t value Pr(>|t|)
## (Intercept)  409.985     37.191  91.796  11.024   <2e-16 ***
## Pretraining   -1.629      2.228  86.621  -0.731    0.467
## ---
## Signif. codes:  0 '***' 0.001 '**' 0.01 '*' 0.05 '.' 0.1 ' ' 1
##
## Correlation of Fixed Effects:
##             (Intr)
## Pretraining -0.919
```

```
model5<-lmer(Observed~Posttraining+ (1|aquarium), data=alphaDiversitySnails)
summary(model5)
```

```
## Linear mixed model fit by REML. t-tests use Satterthwaite's method [
## lmerModLmerTest]
## Formula: Observed ~ Posttraining + (1 | aquarium)
##    Data: alphaDiversitySnails
##
## REML criterion at convergence: 1168.9
##
## Scaled residuals:
##     Min      1Q  Median      3Q     Max
## -1.7680 -0.7035 -0.1683  0.6490  2.6866
##
## Random effects:
##  Groups   Name        Variance Std.Dev.
##  aquarium (Intercept)  2977     54.56
##  Residual             14114    118.80
## Number of obs: 94, groups:  aquarium, 46
##
## Fixed effects:
##              Estimate Std. Error       df t value Pr(>|t|)
## (Intercept)  379.6674    24.9718  82.9137  15.204   <2e-16 ***
## Posttraining   0.4585     1.7529  91.9983   0.262    0.794
## ---
## Signif. codes:  0 '***' 0.001 '**' 0.01 '*' 0.05 '.' 0.1 ' ' 1
##
## Correlation of Fixed Effects:
##             (Intr)
## Posttrainng -0.802
```

```
model6<-lmer(Observed~Alltraining2+ (1|aquarium), data=alphaDiversitySnails) ###t = 0.457    p
=0.649
summary(model6)
```

```
## Linear mixed model fit by REML. t-tests use Satterthwaite's method [
## lmerModLmerTest]
## Formula: Observed ~ Alltraining2 + (1 | aquarium)
##    Data: alphaDiversitySnails
##
## REML criterion at convergence: 1169.6
##
## Scaled residuals:
##     Min      1Q  Median      3Q     Max
## -1.7438 -0.7264 -0.1789  0.5867  2.6285
##
## Random effects:
##  Groups   Name        Variance Std.Dev.
##  aquarium (Intercept)  3034     55.09
##  Residual             14075    118.64
## Number of obs: 94, groups:  aquarium, 46
##
## Fixed effects:
##              Estimate Std. Error       df t value Pr(>|t|)
## (Intercept)  391.3135    35.7014  91.9865  10.961   <2e-16 ***
## Alltraining2  -0.2402     1.2119  88.5899  -0.198    0.843
## ---
## Signif. codes:  0 '***' 0.001 '**' 0.01 '*' 0.05 '.' 0.1 ' ' 1
##
## Correlation of Fixed Effects:
##             (Intr)
## Alltrainng2 -0.908
```

```
##does treatment affect willingness to consume carrot juice?
model3b<-lmer(Pretraining~treatmentOrder+ (1|aquarium), data=alphaDiversitySnails) ###NO
```

```
## boundary (singular) fit: see help('isSingular')
```

```
summary(model3b)
```

```
## Linear mixed model fit by REML. t-tests use Satterthwaite's method [
## lmerModLmerTest]
## Formula: Pretraining ~ treatmentOrder + (1 | aquarium)
##    Data: alphaDiversitySnails
##
## REML criterion at convergence: 590.8
##
## Scaled residuals:
##      Min       1Q   Median       3Q      Max
## -2.35343 -0.71316 -0.02377  0.80552  2.29499
##
## Random effects:
##  Groups   Name        Variance Std.Dev.
##  aquarium (Intercept)  0.00    0.000
##  Residual             36.11    6.009
## Number of obs: 94, groups:  aquarium, 46
##
## Fixed effects:
##                       Estimate Std. Error      df t value Pr(>|t|)
## (Intercept)            15.8571     1.3114 90.0000  12.092   <2e-16 ***
## treatmentOrderlow      -0.5714     1.8546 90.0000  -0.308    0.759
## treatmentOrdermedium  -0.6488     1.7957 90.0000  -0.361    0.719
## treatmentOrderxHigh    -0.7143     1.7348 90.0000  -0.412    0.682
## ---
## Signif. codes:  0 '***' 0.001 '**' 0.01 '*' 0.05 '.' 0.1 ' ' 1
##
## Correlation of Fixed Effects:
##             (Intr) trtmntOrdrl trtmntOrdrm
## trtmntOrdrl -0.707
## trtmntOrdrm -0.730  0.516
## trtmntOrdrH -0.756  0.535       0.552
## optimizer (nloptwrap) convergence code: 0 (OK)
## boundary (singular) fit: see help('isSingular')
```

DMSO effect on behaviour

```
metadataDMSO<-read.csv(file="metadata_DMSO_control.csv",header=TRUE)

model1<-lmer(metabolicRate~ treatmentOrder + (1|aquarium), data = metadataDMSO)
```

```
## boundary (singular) fit: see help('isSingular')
```

```
summary(model1)
```

```
## Linear mixed model fit by REML. t-tests use Satterthwaite's method [
## lmerModLmerTest]
## Formula: metabolicRate ~ treatmentOrder + (1 | aquarium)
##    Data: metadataDMSO
##
## REML criterion at convergence: -31.5
##
## Scaled residuals:
##      Min       1Q   Median       3Q      Max
## -2.15896 -0.56122 -0.00884  0.73184  2.24282
##
## Random effects:
##  Groups   Name        Variance Std.Dev.
##  aquarium (Intercept) 0.00000  0.0000
##  Residual             0.02697  0.1642
## Number of obs: 51, groups:  aquarium, 25
##
## Fixed effects:
##                    Estimate Std. Error       df t value Pr(>|t|)
## (Intercept)         0.38788    0.03501 49.00000  11.078 6.03e-15 ***
## treatmentOrderDMSO  0.07677    0.04643 49.00000   1.654    0.105
## ---
## Signif. codes:  0 '***' 0.001 '**' 0.01 '*' 0.05 '.' 0.1 ' ' 1
##
## Correlation of Fixed Effects:
##             (Intr)
## trtmntODMSO -0.754
## optimizer (nloptwrap) convergence code: 0 (OK)
## boundary (singular) fit: see help('isSingular')
```

```
model2<-lmer(biteRateChange~ treatmentOrder + (1|aquarium), data = metadataDMSO)
summary(model2)
```

```
## Linear mixed model fit by REML. t-tests use Satterthwaite's method [
## lmerModLmerTest]
## Formula: biteRateChange ~ treatmentOrder + (1 | aquarium)
##    Data: metadataDMSO
##
## REML criterion at convergence: 357.5
##
## Scaled residuals:
##     Min      1Q  Median      3Q     Max
## -2.4881 -0.3676  0.1694  0.5522  1.9817
##
## Random effects:
##  Groups   Name        Variance Std.Dev.
##  aquarium (Intercept) 10.68    3.269
##  Residual             66.47    8.153
## Number of obs: 51, groups:  aquarium, 25
##
## Fixed effects:
##                    Estimate Std. Error     df t value Pr(>|t|)
## (Intercept)          -7.754      1.990 24.895  -3.897  0.00065 ***
## treatmentOrderDMSO    1.749      2.682 20.194   0.652  0.52158
## ---
## Signif. codes:  0 '***' 0.001 '**' 0.01 '*' 0.05 '.' 0.1 ' ' 1
##
## Correlation of Fixed Effects:
##             (Intr)
## trtmntODMSO -0.742
```

```
model3<-lmer(Speed~ treatmentOrder + (1|aquarium), data = metadataDMSO)
```

```
## boundary (singular) fit: see help('isSingular')
```

```
summary(model3)
```

```
## Linear mixed model fit by REML. t-tests use Satterthwaite's method [
## lmerModLmerTest]
## Formula: Speed ~ treatmentOrder + (1 | aquarium)
##    Data: metadataDMSO
##
## REML criterion at convergence: 26.2
##
## Scaled residuals:
##     Min      1Q  Median      3Q     Max
## -2.0720 -0.5661 -0.1021  0.5852  2.7125
##
## Random effects:
##  Groups   Name        Variance Std.Dev.
##  aquarium (Intercept) 0.00000  0.0000
##  Residual             0.08754  0.2959
## Number of obs: 51, groups:  aquarium, 25
##
## Fixed effects:
##                     Estimate Std. Error       df t value Pr(>|t|)
## (Intercept)          0.65747    0.06308 49.00000  10.423 4.99e-14 ***
## treatmentOrderDMSO -0.12951    0.08365 49.00000  -1.548    0.128
## ---
## Signif. codes:  0 '***' 0.001 '**' 0.01 '*' 0.05 '.' 0.1 ' ' 1
##
## Correlation of Fixed Effects:
##             (Intr)
## trtmntODMSO -0.754
## optimizer (nloptwrap) convergence code: 0 (OK)
## boundary (singular) fit: see help('isSingular')
```

```
model4<-lmer(Thigmotaxis~ treatmentOrder + (1|aquarium), data = metadataDMSO)
summary(model4)
```

```
## Linear mixed model fit by REML. t-tests use Satterthwaite's method [
## lmerModLmerTest]
## Formula: Thigmotaxis ~ treatmentOrder + (1 | aquarium)
##    Data: metadataDMSO
##
## REML criterion at convergence: 0.8
##
## Scaled residuals:
##     Min      1Q  Median      3Q     Max
## -2.4711 -0.5842  0.1184  0.6963  1.3237
##
## Random effects:
##  Groups   Name        Variance Std.Dev.
##  aquarium (Intercept) 0.008798 0.0938
##  Residual             0.044691 0.2114
## Number of obs: 51, groups:  aquarium, 25
##
## Fixed effects:
##                    Estimate Std. Error       df t value Pr(>|t|)
## (Intercept)         0.57505    0.05292 29.89423  10.867 6.65e-12 ***
## treatmentOrderDMSO -0.06871    0.07150 25.34995  -0.961    0.346
## ---
## Signif. codes:  0 '***' 0.001 '**' 0.01 '*' 0.05 '.' 0.1 ' ' 1
##
## Correlation of Fixed Effects:
##             (Intr)
## trtmntODMSO -0.740
```

Differential Abundance at OTU level

```
rm(list = ls(all.names = TRUE))
library(Maaslin2)
metadata <-read.table("metadata_alphaDiversity.txt", row.names="Row.names", header = TRUE)  ###
this has the scaled data for analyses, example in beta diversity
otus <- read.table("otu-table-centroids-iddef0-400bp.txt")

# OTU differential abundance analysis

fit_data = Maaslin2(
  input_data = otus,
  input_metadata = metadata,
  output = "Maaslin2_output_OTUs_AllFixedTerms",
  fixed_effects = c("treatmentOrder", "scaledBiteRate", "scaledThig", "scaledSpeed", "scaledmet
Rate"),
  random_effects = c("aquarium"),
  reference = c("treatmentOrder,control"),
  plot_scatter = FALSE)


KO <- read.table(file='KO_pred_metagenome_unstrat.tsv', header=TRUE)
#Assigning row names from 1st column
rownames(KO) <- KO[,1]
KO = subset(KO, select = -function. )
KO[] <- lapply(KO, as.integer)
PA <- read.table(file='path_abun_unstrat.tsv', header=TRUE)
#Assigning row names from 1st column
rownames(PA) <- PA[,1]
##remove the duplicate column
PA = subset(PA, select = -pathway )
##make values intergers
PA[] <- lapply(PA, as.integer)

#this metadata file has the full fastq sample names (not the shortened names) that were used in
the picrust analysis.
meta<-read.table(file="metadata_picrust.txt", header=TRUE, row.names = "row.names")
# Edit metadata for downstream analyses: invert the sign (-/+) for biteRateChange
# (more intuitive to interpret where higher values = better memory)


meta$biteRateChange = meta$biteRateChange*(-1)




##scale variables

meta$scaledThig<-scale(meta$Thigmotaxis)
meta$scaledSpeed<-scale(meta$Speed)
meta$scaledBiteRate<-scale(meta$biteRateChange)
meta$scaledmetRate<-scale(meta$metabolicRate)
names(meta)


fit_data2 = Maaslin2(
```

```
  input_data = KO,
  input_metadata = meta,
  output = "Maaslin2_output_KO_AllFixedTerms",
  fixed_effects = c("treatmentOrder", "scaledBiteRate", "scaledThig", "scaledSpeed", "scaledmetRate"),
  random_effects = c("aquarium"),
  reference = c("treatmentOrder,control"),
  plot_scatter = FALSE)

fit_data3 = Maaslin2(
  input_data = PA,
  input_metadata = meta,
  output = "Maaslin2_output_PA_AllFixedTerms",
  fixed_effects = c("treatmentOrder", "scaledBiteRate", "scaledThig", "scaledSpeed", "scaledmetRate"),
  random_effects = c("aquarium"),
  reference = c("treatmentOrder,control"),
  plot_scatter = FALSE)
```

```
sessionInfo()
```

```
## R version 4.2.3 (2023-03-15 ucrt)
## Platform: x86_64-w64-mingw32/x64 (64-bit)
## Running under: Windows 10 x64 (build 19045)
##
## Matrix products: default
##
## locale:
## [1] LC_COLLATE=English_United Kingdom.utf8
## [2] LC_CTYPE=English_United Kingdom.utf8
## [3] LC_MONETARY=English_United Kingdom.utf8
## [4] LC_NUMERIC=C
## [5] LC_TIME=English_United Kingdom.utf8
##
## attached base packages:
## [1] stats     graphics  grDevices utils     datasets  methods   base
##
## other attached packages:
##  [1] MuMIn_1.47.5         usdm_1.1-18          raster_3.6-20
##  [4] sp_1.6-1             compositions_2.0-6   metagenomeSeq_1.40.0
##  [7] RColorBrewer_1.1-3   glmnet_4.1-7         limma_3.54.2
## [10] Biobase_2.58.0       BiocGenerics_0.44.0  lmerTest_3.1-3
## [13] lme4_1.1-33          Matrix_1.5-3         phyloseq_1.42.0
## [16] lubridate_1.9.2      forcats_1.0.0        stringr_1.5.0
## [19] dplyr_1.1.2          purrr_1.0.1          readr_2.1.4
## [22] tidyr_1.3.0          tibble_3.2.1         ggplot2_3.4.2
## [25] tidyverse_2.0.0      vegan_2.6-4          lattice_0.20-45
## [28] permute_0.9-7
##
## loaded via a namespace (and not attached):
##  [1] minqa_1.2.5            colorspace_2.1-0       XVector_0.38.0
##  [4] rstudioapi_0.14        fansi_1.0.4            codetools_0.2-19
##  [7] splines_4.2.3          cachem_1.0.8           robustbase_0.95-1
## [10] knitr_1.43             ade4_1.7-22            jsonlite_1.8.4
## [13] nloptr_2.0.3           cluster_2.1.4          compiler_4.2.3
## [16] fastmap_1.1.1          cli_3.6.1              htmltools_0.5.5
## [19] tools_4.2.3            igraph_1.4.2           gtable_0.3.3
## [22] glue_1.6.2             GenomeInfoDbData_1.2.9 reshape2_1.4.4
## [25] Rcpp_1.0.10            jquerylib_0.1.4        vctrs_0.6.1
## [28] Biostrings_2.66.0      rhdf5filters_1.10.1    multtest_2.54.0
## [31] ape_5.7-1              nlme_3.1-162           iterators_1.0.14
## [34] tensorA_0.36.2         xfun_0.39              timechange_0.2.0
## [37] lifecycle_1.0.3        gtools_3.9.4           terra_1.7-29
## [40] DEoptimR_1.0-14        zlibbioc_1.44.0        MASS_7.3-58.2
## [43] scales_1.2.1           hms_1.1.3              parallel_4.2.3
## [46] biomformat_1.26.0      rhdf5_2.42.1           yaml_2.3.7
## [49] sass_0.4.6             stringi_1.7.12         highr_0.10
## [52] S4Vectors_0.36.2       foreach_1.5.2          caTools_1.18.2
## [55] boot_1.3-28.1          shape_1.4.6            GenomeInfoDb_1.34.9
## [58] rlang_1.1.0            pkgconfig_2.0.3        bitops_1.0-7
## [61] matrixStats_1.0.0      Wrench_1.16.0          evaluate_0.21
## [64] Rhdf5lib_1.20.0        tidyselect_1.2.0       plyr_1.8.8
## [67] magrittr_2.0.3         R6_2.5.1               IRanges_2.32.0
## [70] gplots_3.1.3           generics_0.1.3         DBI_1.1.3
## [73] pillar_1.9.0           withr_2.5.0            mgcv_1.8-42
## [76] survival_3.5-5         RCurl_1.98-1.12        bayesm_3.1-5
```

```
## [79] crayon_1.5.2           KernSmooth_2.23-21     utf8_1.2.3
## [82] tzdb_0.4.0             rmarkdown_2.22         locfit_1.5-9.8
## [85] grid_4.2.3             data.table_1.14.8      digest_0.6.31
## [88] numDeriv_2016.8-1.1    stats4_4.2.3           munsell_0.5.0
## [91] bslib_0.5.0
```
